# Framing fact-checks as a “confirmation” increases engagement with corrections of misinformation: a four-country study

**DOI:** 10.1038/s41598-024-53337-0

**Published:** 2024-02-08

**Authors:** Natalia Aruguete, Flavia Batista, Ernesto Calvo, Matias Guizzo-Altube, Carlos Scartascini, Tiago Ventura

**Affiliations:** 1 Departamento de Ciencias Sociales, Universidad Nacional de Quilmes UNQ, 1876 Bernal, Buenos Aires Argentina; 2 Department of Government and Politics, University of Maryland UMD, College Park, MD 20742 USA; 3 Research Department, Inter-American Development Bank IDB, Washington, DC 20577 USA; 4 McCourt School of Public Policy, Georgetown University, Washington, DC 20057 USA

**Keywords:** Psychology, Human behaviour

## Abstract

Previous research has extensively investigated why users spread misinformation online, while less attention has been given to the motivations behind sharing fact-checks. This article reports a four-country survey experiment assessing the influence of *confirmation* and *refutation* frames on engagement with online fact-checks. Respondents randomly received semantically identical content, either affirming accurate information (“It is TRUE that *p*”) or refuting misinformation (“It is FALSE that *not p*”). Despite semantic equivalence, confirmation frames elicit higher engagement rates than refutation frames. Additionally, confirmation frames reduce self-reported negative emotions related to polarization. These findings are crucial for designing policy interventions aiming to amplify fact-check exposure and reduce affective polarization, particularly in critical areas such as health-related misinformation and harmful speech.

## Introduction

Fact-checking is today the first line of defense against misinformation^1–4^. It is frequently defined as “the practice of systematically publishing assessments of the validity of claims made by public officials and institutions with an explicit attempt to identify whether a claim is factual”^5^, p. 350. Research shows that fact checks successfully influence people’s discernment of misinformation and *nudge* users to update their beliefs after correction, whether in survey experiments or field experiments, and across different cultural contexts^1,6–8^. The effect of fact-checking interventions extends over time, with minimal evidence of backfire effects from exposure to fact-checking corrections^9,10^.

To curb the spread of misinformation, fact-checkers can employ two distinct framing strategies: they can either publish *confirmation frames* that replace misinformation with accurate information, or they can publish *refutation frames* that warn social media users about content tagged as misinformation^11^. Choosing *confirmations* provides users with factually accurate content they can share with peers. Opting for *refutations* allows fact-checkers to decrease the sharing of inaccurate, misleading, or false content. The effectiveness of increasing “good” content versus reducing “bad” content has not been experimentally tested. In this paper, we evaluate the impact of *confirmation* and *refutation* frames on the sharing behavior of social media users.

The lack of studies measuring the impact of *confirmation (TRUE)* and *refutation (FALSE)* frames is surprising, given the central role content labeling plays in fact-checking interventions. As noted by Shin and Thorson, “[u]nlike traditional journalism, which emphasizes detached objectivity and adheres to the ‘he said, she said’ style of reporting, contemporary fact-checking directly engages in adjudicating factual disputes by publicly deciding whose claim is correct or incorrect”^12^, p. 1. The decision to intervene using *confirmation* or *refutation* frames is an editorial choice that is independent of the source material^13^.

This paper presents experiments conducted in four different countries to assess the effect of *confirmation* and *refutation* frames on the sharing behavior of social media users. Our experiments expose nationally representative samples of Argentine, Brazilian, Chilean, and Colombian respondents to edited Facebook posts framed as a confirmation of accurate information or a refutation of misinformation. The experiment rotates the confirmation and refutation frames, the choice of labels (labeled vs. unlabeled), and the type of vaccine (Moderna, AstraZeneca, and Sputnik V). The variation in vaccines only takes place in Argentina. The empirical analysis and robustness checks include several control variables (i.e., socio-demographic, attitudinal, and health status variables) and validation checks (i.e., processing time and pseudo-placebo treatment).

Our primary outcome measures the decision to engage (i.e., “like,” “share,” and “comment”) with the fact check and the self-reported affective response to the fact-checking post. Our hypotheses, pre-registered at https://osf.io/ prior to the collection of the data, posit that respondents will engage more with confirmation frames than refutation frames [Hypothesis 1 (H1)]. We propose this effect to be independent of other factors prompting engagement with a correction, such as cognitive congruence and partisan attachment. Our primary hypothesis stems from two theoretical mechanisms: the heavier cognitive burden of refutation frames and the positive valence charge associated with confirmation frames. We offer specific hypotheses and dedicated tests for each mechanism.

First, negation imposes a heavier cognitive load^14^. Research in cognitive linguistics and cognitive psychology has documented differences in processing semantically equivalent positive or negative statements. Kaup, Lüdtke, and Zwaan show that individuals are faster to process statements such as “the umbrella was open” compared to its semantically equivalent “the umbrella was not closed”^15^. Subjects also display faster response times for “the umbrella was closed” than for “the umbrella was not open.” Indeed, this cognitive effort is not the result of the state of the umbrella (i.e., *open* or *closed*), but the result of how we process negation statements. In social networks, a higher cognitive burden could conceivably deter a swift, automatic, and affective response^16,17^, leading to more evaluative sharing behavior. We hypothesize that refutation frames exert a higher cognitive burden on respondents, thus resulting in longer reading times [Hypothesis 2 (H2)] that curtail sharing.

Second, we expect that the confirmation of pro-attitudinal beliefs will carry a positive valence charge compared to the refutation of a counter-attitudinal belief. A standard sentiment analysis using state-of-the-art RoBERTa^18^ shows that “It is true that vaccines are effective” is classified as *Positive* (i.e., Cardiff scores are Positive: 0.782, *Neutral*: 0.209, *Negative*: 0.009). Meanwhile, “It is false that vaccines are not effective” is classified as *negative* (i.e., Cardiff scores are *Positive*: 0.024, *Neutral*: 0.278, *Negative*: 0.698). This is because the words “true” and “false” function not only as Boolean operators but also convey positive and negative connotations in social conversation.

Confirmation statements such as “it is TRUE that *p*” convey that the content is socially acceptable and less likely to expose users to public scrutiny and criticism. Tetlock coins the term “intuitive politician” to describe the behavior of risk-averse subjects who seek to preserve their reputation by aligning themselves with socially accepted positions^19^. “People behave like intuitive politicians when they seek to maintain a positive reputation or fulfill the social duties for which they are accountable”^20^. Therefore, confirmation frames communicate greater social acceptability and widespread consensus with published content.

Refutation frames, in contrast, suggest that there are dissenting opinions and raise the potential for conflict. That is, refutation frames suggest that at least some individuals or groups have competing beliefs^19^. Therefore, statements framed as confirmations will have a positive valence charge that is independent of the pro- or counter-attitudinal preferences for the denoted content in the message. We hypothesize that confirmation frames will elicit positive emotional reactions and refutations will elicit negative ones [Hypothesis 3 (H3)].

To sum up our pre-registered hypotheses, we anticipate the statement “it is TRUE that p” to enhance engagement compared to “it is FALSE that not p” [Hypothesis 1 (H1)], because the former is both cognitively simpler to process [Hypothesis 2 (H2)], and because *TRUE* carries an inherent positive valence charge [Hypothesis 3 (H3)]. Conversely, refutation statements are cognitively challenging, and sharing refutation messages aligns one with an in-group social media user at odds with an out-group user’s beliefs.

### From theory to design

**Figure 1:**
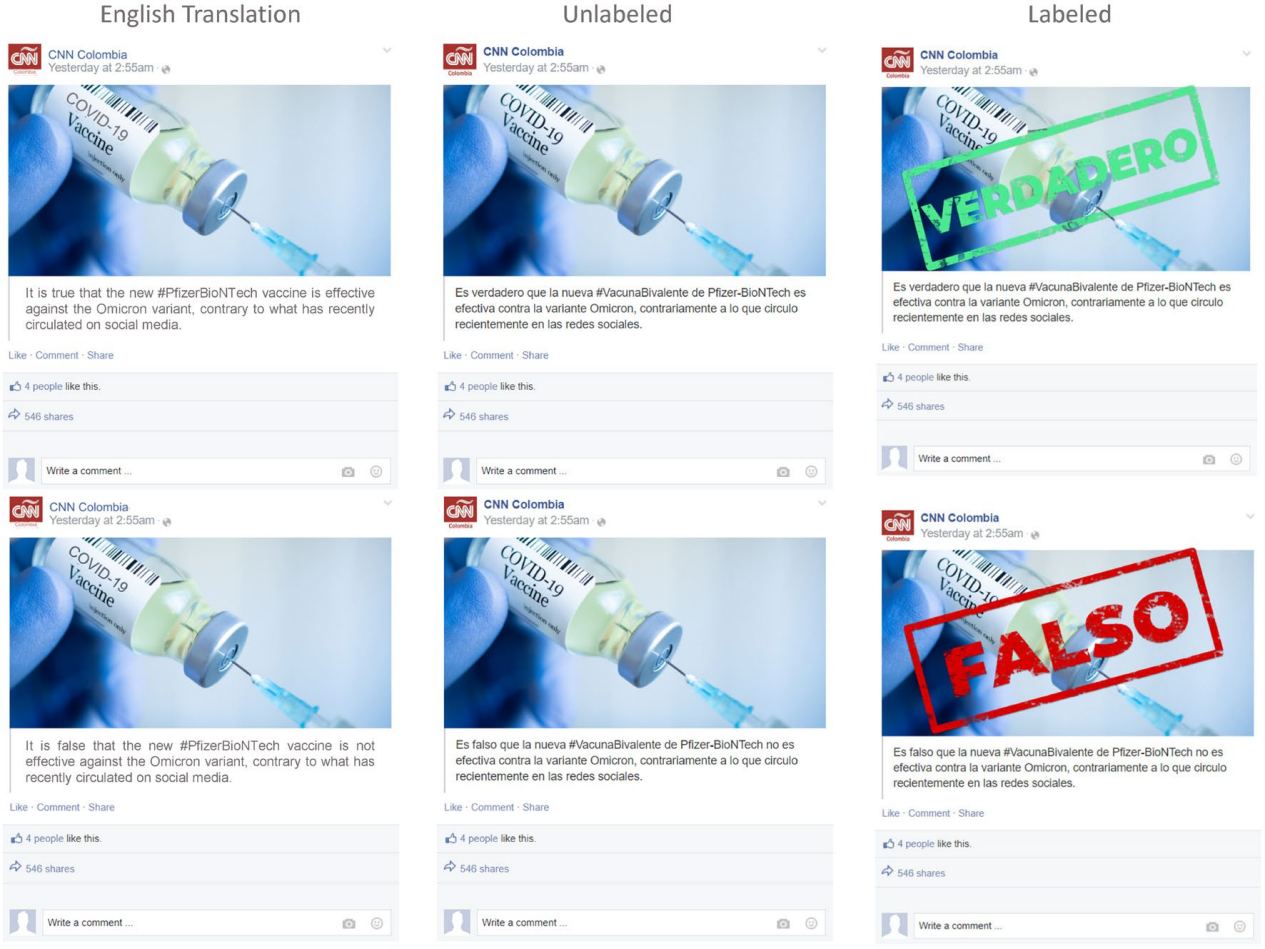
Images of the *Confirmation* (“It is TRUE that p”) and *Refutation* (“It is FALSE that not p”) treatments used in Colombia, with English translations for clarity. While semantically equivalent, the confirmation and refutation frames differ in cognitive accessibility and valence charge. All treatments are factually accurate and align with the design employed by our partner organization in Argentina, *Chequeado*. The designs for each country, including the placebo, are detailed in the Supplemental Information File accompanying this article.

The two-arm design exposes respondents to a Facebook post that randomly confirms a clinically correct statement or refutes a clinically incorrect statement. Crucially, the experiment did not spread misinformation to participants; both the *confirmation* and the *refutation* frames communicated that vaccines are effective against the Omicron variant. The decision to use clinically accurate information also prevents us from testing the effect of the logically equivalent statements “It is TRUE that not-p” and “It is FALSE that p”. We discuss this limitation in our concluding remarks. In Argentina, we implement three different Facebook post designs with the Sputnik V, Moderna, or AstraZeneca vaccines. In Chile, Brazil, and Colombia, we implement two distinct designs, presenting *confirmation* or *refutation* treatments with explicit labels or without labels (Fig. 1 presents the Colombia treatments. See the complete set of treatments in the Supplementary Information File (SIF), Figs. S1, S2, S3, and S4).

Additionally, we introduced confirmation and refutation frames unrelated to our health correction and devoid of any correlation with political preferences. This pseudo-placebo treatment measures the independent valence charge associated with using the words “true” and “false” in a post about dogs.

In all four countries, simple randomization was implemented, with respondents having equal chances of being assigned to each treatment (*confirmation* or *refutation* of the vaccines or dog treatments) and to each of the design alternatives (label, no-label, and, in the case of Argentina, vaccine type—Table S1 through Table S4 in the SIF present summary statistics and balance across the treatments).

After exposure to the treatments, respondents were asked to indicate whether they would “like,” “share,” and/or “comment” on the Facebook post. The response format allowed for multiple selections, with an explicit “ignore” option that was exclusive if chosen. Additionally, participants were asked to self-report their emotional response to the post, choosing from a list that included Ekman’s six basic emotion categories: *fear*, *anger*, *joy*, *sadness*, *disgust*, and *surprise*, as well as an additional positive category, *optimism*. Multiple responses were allowed, except for the alternative *indifferent*, which was exclusive if selected.

The sequence of presentation (the Facebook treatments, the sharing behavior, and the emotional response) remained the same for all survey respondents. Additionally, we recorded the time-to-read (the elapsed time spent viewing the post), the time-to-react (the elapsed time before responding to the behavior question), and the time-to-feel (the elapsed time before reporting an emotional reaction). The survey collected additional information to allow the inclusion of various demographic, political, and COVID-19 risk factors in the empirical analysis.

## Methodology

### Survey design

The “vaccine” experiments use a two-arm design that exposes respondents to one of two equivalent statements that *confirm* the efficacy of the vaccine or refute its inefficacy. The design randomly prompts respondents to read either the confirmation statement “*It is TRUE that the new #VacunaBivalente is effective against the Omicron variant*” or the refutation of the corresponding misinformation “*It is FALSE that the new #VacunaBivalente is not effective against the Omicron variant*.” In Argentina, the vaccine brands (Sputnik V, Moderna, and AstraZeneca) are rotated. In Brazil, Chile, and Colombia, the use of labels is rotated, and a pseudo-placebo treatment about dogs is included.

The flow of the experiment is as follows. First, respondents are exposed to either a *confirmation* or *refutation* frame. We measure the time respondents spend reading the statement (time-to-read), beginning with the image loading and ending when the respondent progresses to the next page of the online survey. The second page asks respondents if they would “like”, “share”, “comment”, or “ignore” the Facebook post. We measure the time-to-respond. Finally, respondents are asked to self-report their emotional reaction to the question on the third page.

The statistical models employ simple two-tailed mean tests. The conditional effects of time variables and other socio-economic indicators are further assessed using general linear regression and ordinary least-square models.

### Survey descriptive information

The survey experiments were conducted in Argentina in February 2022, Chile in November 2022, Brazil in December 2022, and Colombia in March 2023. The surveys were designed by the Interdisciplinary Lab for Computational Social Science (iLCSS) at the University of Maryland, College Park, in collaboration with the Fact-Checking Agency *Chequeado*.

All four surveys were administered online by the polling firm Netquest, recruiting respondents from opt-in panels in each country and using quota sampling to achieve nationally representative samples on key demographics, such as age, gender, population, and income. An Independent assessment of the quality of Netquest panels compared with a probabilistic sample was recently published by Castorena et al.^21^, finding very small deviations from optimal sampling. The total number of respondents of the vaccine experiments comprised 9512 adult respondents from Argentina (2349), Brazil (2401), Chile (2384), and Colombia (2378), with stratification based on age, education, gender, and region in accordance with current census data. The survey took a median time of 22 minutes to complete. In addition to the experiment, it included a battery of socio-demographic, attitudinal, and political questions. Table 1 presents descriptive statistics for selected variables in each country sample. For robustness, we created survey weights to correct minor sampling deviations in education and gender. Tests show that sampling assignments were balanced across treatments, and sampling weights have no substantive effect on the experiment results.

### Variable definitions

#### Dependent variables

We test for the effect of confirmation and refutation frames on four key behavioral responses and seven self-reported emotions to the treatments. After seeing the Facebook Post, respondents were asked to “like,” “share,” “comment,” or “ignore” it. Each reaction is treated as a dependent variable. In addition, there is an indicator variable (engage) for selecting at least one active reaction (like, retweet, or reply) by the respondent.

After seeing the Facebook Post, respondents were also asked if the publication elicited the following emotions: Anger, contempt, disgust, optimism, stress, sadness, fear, or indifference. Respondents could mark more than one option. For our analyses, each emotion is associated with one indicator variable and is treated as a single dependent variable.

**Table 1:** Descriptive statistics by country.

Variable	Argentina (N = 2349)	Brazil (N = 2401)	Chile (N = 2384)	Colombia (N = 2378)
Age (years)	40.48 (12.59)	39.61 (12.03)	42.03 (12.43)	37.55 (12.11)
Educational attainment				
Incomplete secondary (or −)	5.75% (23.28)	9.08% (28.74)	1.59% (12.53)	2.99% (17.02)
Completed secondary	19.67% (39.76)	28.53% (45.17)	19.63% (39.73)	21.4% (41.02)
Incomplete college	32.14% (46.71)	14.79% (35.5)	21.52% (41.1)	23.76% (42.57)
Completed college	35.46% (47.85)	27.41% (44.61)	46.39% (49.88)	40.71% (49.14)
Incomplete graduate (or +)	6.98% (25.49)	20.2% (40.16)	10.86% (31.13)	11.14% (31.47)
Woman	56.11% (49.64)	49.52% (50.01)	57.72% (49.41)	53.66% (49.88)
Employed	79.99% (40.01)	75.82% (42.82)	79.23% (40.57)	82.25% (38.22)
Vote for incumbent party	25.71% (43.71)	38.15% (48.59)	42.2% (49.4)	48.91% (50)
Vote for opposition party	44.44% (49.7)	42.69% (49.47)	34.77% (47.64)	18.29% (38.67)
Time to read (log of seconds)	2.81 (0.79)	2.77 (0.74)	2.97 (0.73)	2.96 (0.74)
Have had COVID-19	43.85% (49.63)	44.94% (49.75)	40.06% (49.01)	42.8% (49.49)
Vaccines against COVID-19				
Non-vaccinated against COVID-19	6.02% (23.8)	5.14% (22.08)	4.5% (20.73)	6.16% (24.05)
Vaccinated once	3.12% (17.38)	3.59% (18.61)	1.3% (11.34)	9.96% (29.95)
Vaccinated twice (or +)	90.86% (28.83)	91.27% (28.23)	94.2% (23.38)	83.88% (36.78)

Coefficients represent the average value of each control variable in the complete sample from each country survey. Standard deviations in parentheses.

#### Treatment variables

The most important independent variable is binary, indicating if the respondent was exposed to the *confirmation* frame (“It is TRUE that p”) or the refutation frame (“It is FALSE that not p”). We also control for different treatment designs: in Argentina, an indicator variable for the vaccine brand mentioned in the vignette (AstraZeneca, Moderna, or Sputnik V). In Brazil, Chile, and Colombia, an indicator variable describing if respondents were treated to the post with no labels, the post with labels, or the “Dog” treatment.

#### Control variables

For completeness, we estimate restricted models with only the treatment variables and unrestricted models with several important controls. These controls, described in Table 1, include a measure of cognitive difficulty describing the time in seconds (log) spent by the respondent reading the treatment; a set of education indicator variables (i.e., elementary, middle/high school education, undergraduate, and graduate degrees); binary variables indicating vote intention for the incumbent and opposition parties “if the election were to take place next week”; variables for six age groups (i.e., 18–25, 26–35, 36–45, 46–55, 56–65, and more than 65 years of age); a variable indicating if the respondent is a woman; a variable indicating if the respondent “is currently employed;” a variable indicating if the respondent ever contracted COVID-19; and, finally, variables for the number of COVID-19 vaccine doses.

### Ethics

Human subjects and ethics approval was granted by the University of Maryland Institutional Review Board before the implementation of surveys in each country. The project approvals are registered under the identification code IRB 1825785, beginning with IRB [1825785-1] “COVID-19, Trust, and Misinformation,” approved on October 27, 2021. Further approvals for each survey are registered under the identification codes 1825785-2 through [1825785-8], with final approval on January 19, 2023. Decisions by the review board granted all four surveys expedited review category #7. Waiver of Consent Documentation, 45CFR46.117(c). Waiver of Consent 45CFR46.116(f)(3) (deception) was granted as we exposed respondents to treatments created by our research team. A disclaimer provided respondents with information on contacting the researchers or IRB if needed. Informed consent was requested on the “start page” of the online survey. Once the survey was completed, consent to use the data was again requested in the exit question. The survey was not executed without explicit consent on the opening page, and the survey was not stored without explicit consent at the end of the survey.

### Methods

All methods were carried out in accordance with relevant guidelines and regulations.

## Results

**Figure 2:**
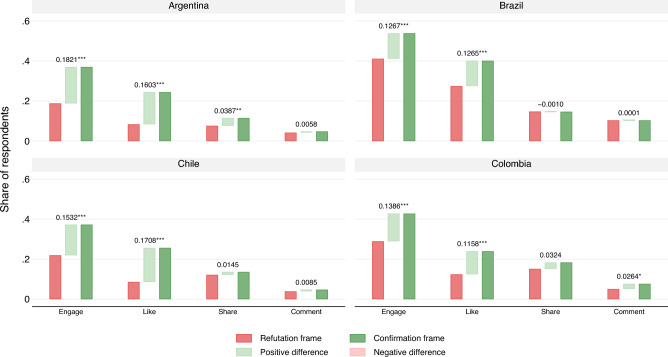
This figure displays the four-country regression results for “engagement,” “like,” “share,” and “comment”. A trio of bars represents each dependent variable: the first red bar shows the result for the refutation frame, while the third green bar presents the result for the confirmation frame. The middle bar shows the difference between the refutation and confirmation frames. A light green indicates a positive difference (confirmation frames eliciting more engagement than refutation), and a light red indicates a negative difference. We report the numeric difference between the two frames and their two-tailed significance, ***p < 0.001, **p < 0.01, *p < 0.05, with robust standard errors. See SIF documentation for full regression models with and without controls.

*Confirmation* frames led to higher engagement than *refutation* frames across the four countries involved in the study, supporting Hypothesis 1 (H1). Detailed results of the restricted models (without controls) are shown in Fig. 2, and the difference between the frames for the unrestricted models with two-tailed tests and standard errors are reported in Table 2. Full models and robustness checks for all four countries are reported in Tables S5, S6, S7, and S8 of the SIF accompanying this article. In Argentina, engagement rose from 0.189 (or 18.9% of combined likes, shares, and comments) to 0.371 (or 37.1%), a substantive difference of 0.182 ($SE=0.018$), equivalent to a twofold increase of 18.2 p.p. (percentage points). The increase is statistically significant, with a t-value of 10.1 ($p<0.001$). The differences in engagement for Brazil, Chile, and Colombia are also large in magnitude (13, 15, and 14 percentage points, respectively) and statistically significant at the $p<0.001$ level.

The difference in “likes” is large and statistically significant in all four countries. Confirmation frames increase reported likes by 16 p.p. in Argentina, 13 p.p. in Brazil, 17 p.p. in Chile, and 12 p.p. in Colombia. The threefold increase in Argentina and Chile and the twofold increase in Brazil and Colombia are statistically significant at the $p<0.001$ level. The effect of the confirmation frame is more modest and less consistent in explaining the decision to share and comment on the Facebook posts. Only in Argentina ($p<0.01$) is the increase in sharing statistically significant. Similarly, The increase in reported “comment” is significant only in Colombia ($p<0.05$) and small in magnitude.

**Figure 3:**
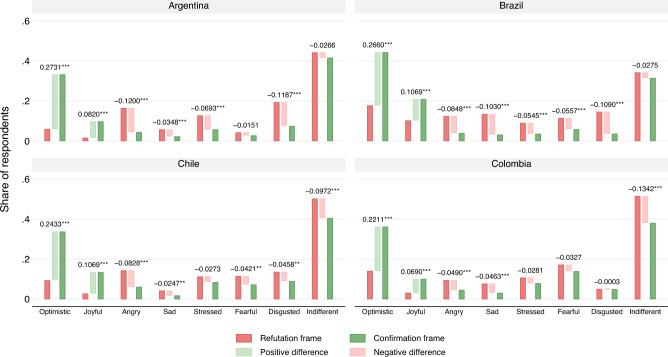
This figure displays the four-country regression results for self-reported emotion. A trio of bars represents each dependent variable: the first red bar shows the result for the refutation frame, and the third green bar presents the result for the confirmation frame. The middle bar shows the difference between the refutation and confirmation frames. We report the numeric difference between the two frames and their two-tailed significance, ***p < 0.001, **p < 0.01, *p < 0.05, with robust standard errors. See SIF documentation for full regression models with and without controls.

The effect of the confirmation and refutation frames on reported emotion [Hypothesis 3 (H3)] is consistent with our expectations. As illustrated in Fig. 3, individuals exposed to the confirmation frame reported significantly more “joyful” and “optimistic” responses, significant at the $p<0.001$ level. These differences are quite pronounced, ranging from a more than two-fold increase in reported optimism and joy in Brazil ($p<0.001$) to more than a five-fold increase in Argentina($p<0.001$). In contrast, the refutation frame was primarily linked with negative emotions, such as “anger,” ($p<0.001$) “disgust,” (significant at $p<0.001$ in Argentina and Brazil, and $p<0.01$ in Chile), and “stress” (significant at $p<0.001$ in Argentina and Brazil). The effects were substantively and statistically significant. For instance, in Argentina, the refutation frame was four times more likely to induce anger than the confirmation frame. Similarly, the refutation frame was at least twice as likely to elicit anger in Brazil, Chile, and Colombia.

Table 2 presents the difference between the confirmation and refutation frames for all four countries. The complete set of results can be found in Table S5 through Table S8 in the Supplemental Information File (SIF). The SIF also includes extensive robustness checks, alternative estimates with and without controls, heterogeneous effects for socio-demographic and attitudinal questions, and heterogeneous effects by party. Results underline the consistency of the framing effects, with comparable estimates for the confirmation and refutation frames across countries.

Across all four countries, Table 2 consistently indicates that the emotional responses to the treatments are more positive for the confirmation frames and more negative for the refutation frames. The confirmation frame elicits feelings of optimism and joyfulness, according to the self-reported emotions of respondents. On the other hand, the refutation frame consistently provokes more negative emotions, such as anger, sadness, stress, fear, and disgust. Even though the refutation frame communicates information semantically equivalent to the confirmation frame, it consistently evokes stronger negative emotions. The finding that refutation frames consistently elicit negative affective responses is an important finding for studying affective polarization, a phenomenon well-documented in the existing literature.

**Table 2:** Difference of means between the *confirmation* and *refutation* frames.

Variable	Argentina	Brazil	Chile	Colombia
Reactions				
Engage	0.188*** (0.018)	0.131*** (0.024)	0.152*** (0.023)	0.147*** (0.024)
Like	0.163*** (0.015)	0.127*** (0.022)	0.171*** (0.018)	0.120*** (0.020)
Share	0.042*** (0.012)	0.001 (0.018)	0.010 (0.017)	0.035 (0.019)
Comment	0.008 (0.009)	−0.002 (0.015)	0.006 (0.010)	0.026* (0.012)
Emotions				
Optimistic	0.278*** (0.015)	0.258*** (0.022)	0.246*** (0.020)	0.226*** (0.022)
Joyful	0.087*** (0.009)	0.106*** (0.018)	0.105*** (0.014)	0.071*** (0.013)
Angry	−0.121*** (0.012)	−0.083*** (0.014)	−0.081*** (0.015)	−0.049*** (0.013)
Sad	−0.035*** (0.008)	−0.102*** (0.014)	−0.023** (0.009)	−0.049*** (0.011)
Stressed	−0.072*** (0.012)	−0.051*** (0.012)	−0.025 (0.015)	−0.028 (0.015)
Fearful	−0.015 (0.008)	−0.051*** (0.014)	−0.042** (0.015)	−0.037* (0.019)
Disgusted	−0.121*** (0.014)	−0.112*** (0.014)	−0.046** (0.015)	0.001 (0.011)
Indifferent	−0.028 (0.020)	−0.029 (0.023)	−0.102*** (0.025)	−0.133*** (0.025)

Robust standard errors in parentheses. ***p < 0.001, **p < 0.01, *p < 0.05. Each cell corresponds to a different regression using as sample the survey of the country indicated in the header. Coefficients represent the effect of the confirmation frame on the reaction or emotion indicated in the first column compared against the refutation frame. All regressions control for age, sex, educational attainment, employment status, partisan attachment, having had COVID-19, number of doses administered of COVID-19 vaccine, and time spent reading the post. Full set of models in the SIF file to this article.

**Figure 4:**
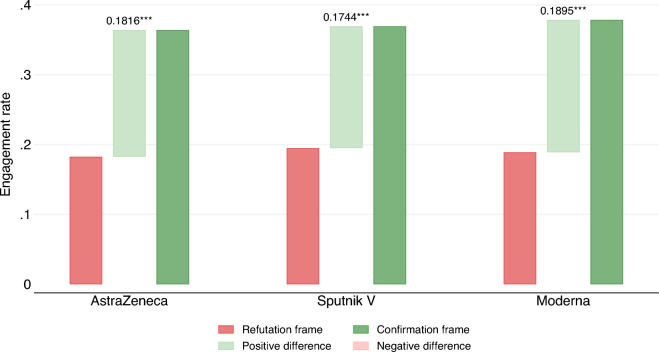
Argentine experiment: overall “engagement” (like $+$ share $+$ comment) using the confirmation and refutation frames, TRUE or FALSE alternatively. Separate means are presented for each vaccine brand: AstraZeneca, Sputnik V, and Moderna. The TRUE and FALSE statements are semantically identical but differ in their cognitive accessibility and their valence charge. Both the TRUE and FALSE adjudications are factually correct.

We now describe the findings for the different types of treatments in Argentina, Brazil, Chile, and Colombia. Figure 4 shows the average rates of engagement (i.e., the sum of “like,” “share,” and “comment” rates) across different brands of the COVID-19 vaccine available in Argentina at the time of the survey, AstraZeneca, Sputnik V, and Moderna. Despite their reduced samples, engagement rates are statistically indistinguishable across brands and remain significant at the $p<0.001$ level. This is especially noteworthy in Argentina, where vaccine brands became a politically charged issue. Indeed, our survey shows that government supporters “engaged” with the Sputnik V vaccine at higher rates than opposition supporters. However, the differences between the confirmation and refutation frames held constant within parties and across brands. Such consistency emphasizes the robustness of the framing effect. The Supplemental Information File comprehensively describes the findings by party, brand, and for each behavior separately (e.g., “like,” “share,” and “comment”).

**Figure 5:**
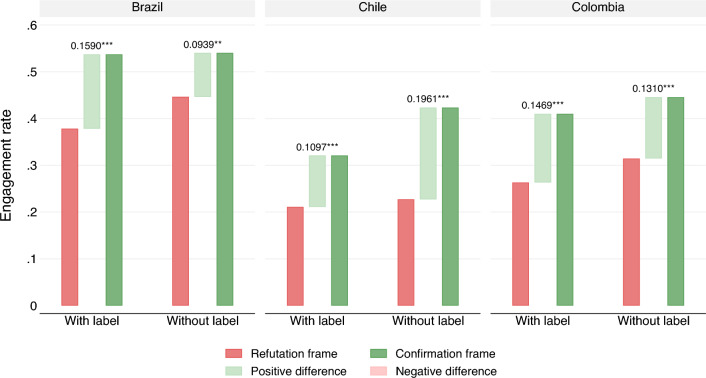
“Engagement” rate using the confirmation and refutation frames, TRUE or FALSE alternatively. Separate means are presented for the treatments with and without explicit labels. The TRUE and FALSE statements are semantically identical but differ in their cognitive accessibility and their valence charge. Both the TRUE and FALSE adjudications are factually correct.

Figure 5 presents the results for Brazil, Chile, and Colombia, distinguishing treatments with and without labels (labels refer to the large banners placed over the picture, as depicted in Fig. 1). Whether to use labels is one of the most heavily discussed topics among fact-checkers^7,22^. A review of existing research suggests that explicit labels reduce trust in misinformation and the correction of misinformation, thereby reducing the likelihood that content will be shared^7^. This raises the possibility of heterogeneous effects across the confirmation and refutation frames.

Three results are worth highlighting when comparing the effect of confirmation and refutation frames with and without labels. First, confirmation frames increase engagement and elicit the expected affective responses for each subsample at the $p<0.001$ level, with the exception of the Brazilian subsample without labels, which shows significance at the $p<0.01$ level. Therefore, *H*1 and *H*3 are confirmed for all three countries for each treatment. Second, consistent with Clayton et al., labels reduce engagement^7^. It is easy to observe in Fig. 5 that the refutation frames with labels elicit lower engagement than the refutation frames with labels. Similarly, confirmation frames with labels elicit lower engagement than confirmations without labels. Therefore, labels reduce overall engagement. However, the magnitude of the difference between the confirmation and refutation frames is not consistent across all three countries. In Brazil and Colombia, the difference between the confirmation and refutation frames is larger for the unlabeled treatments. In Chile, in contrast, the difference between the confirmation and refutation frames is larger for the labeled treatment. Therefore, while all hypotheses hold for the smaller subsamples and we effectively validate the study by Clayton et al., the magnitude of the framing effect for labeled and unlabeled treatments varies across countries^7^. The complete analyses of the heterogeneous effects by country are reported in the supplemental information file.

### Beyond vaccines: dogs do not understand what we say to them

**Figure 6:**
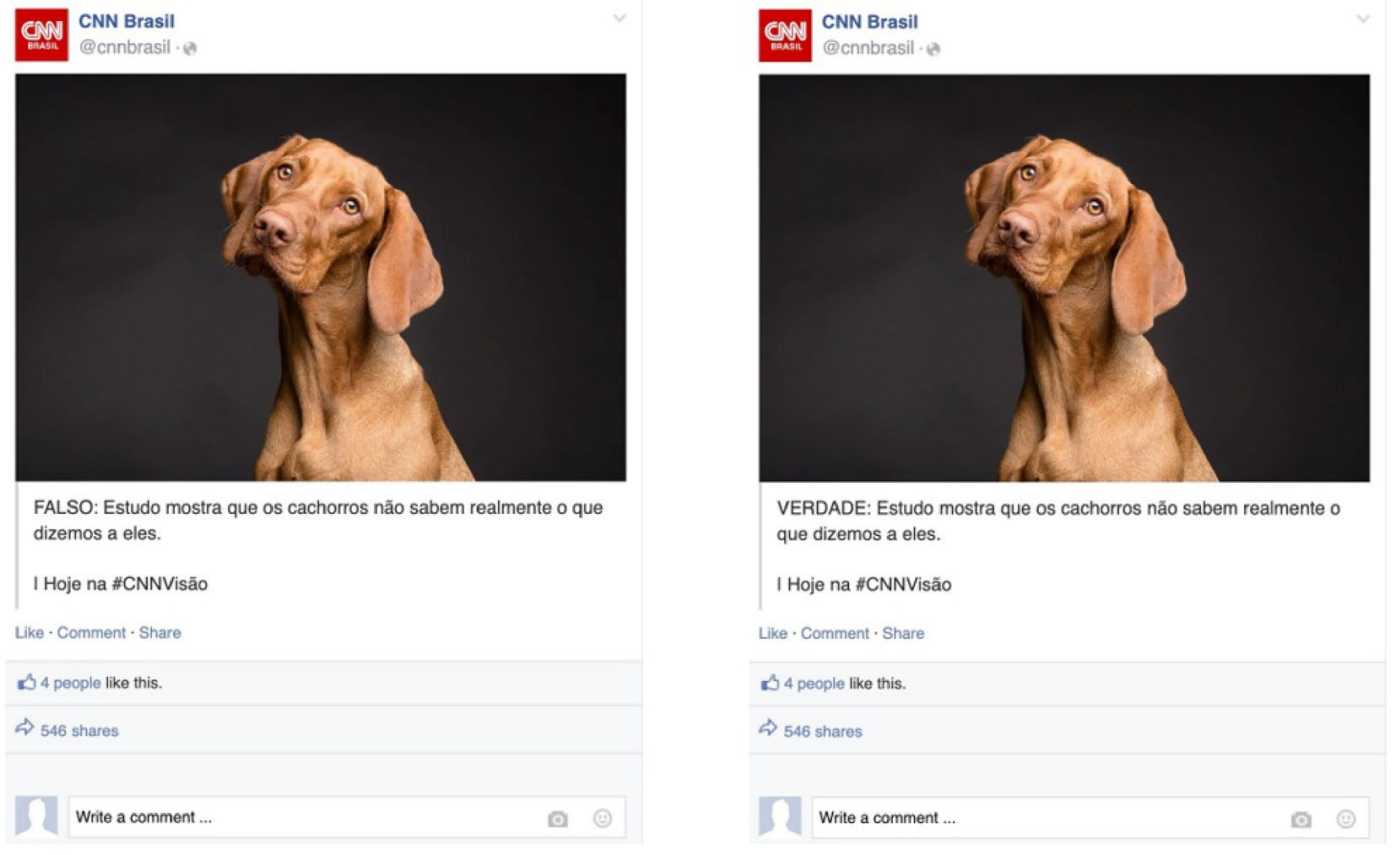
Images of the *confirmation* (“It is TRUE that p”) and *refutation* (“It is FALSE that not p”) pseudo-placebo treatments used in Brazil. The confirmation and refutation frames are semantically equivalent and intended to be equivalent in their cognitive accessibility and their valence charge, changing only the word “True” for “False.” Both treatments conform to the design used by our partner organization in Argentina, *Chequeado*. The pseudo-placebo designs for each country are reported in the Supplemental Information File of this article.

In Brazil, Chile, and Colombia, we included a non-vaccine-related treatment to test the robustness of our findings. The alternative treatment exposed survey participants to a minimally modified CNN post framed either as a confirmation (“True: Dogs do not really understand what we say to them”) or as a refutation (“False: Dogs do not really understand what we say to them”). The treatment avoided the double negative statement by using a colon “:” after “True” or “False”. Qualitative assessment with Spanish speakers validates that the two phrases are interpreted as semantically equivalent. Respondents understand the confirmation as a validation that dogs do not understand what we say. In contrast, respondents understand the refutation as rejecting prior information and affirming that dogs do not understand what we say. Figure 6 exemplifies the treatments using images from the Brazilian survey. The only difference between the two treatments is the inclusion of the words “True:” or “False:”. This design ensures that the same content is conveyed while maintaining consistency in the message’s semantic meaning and cognitive complexity.

**Figure 7:**
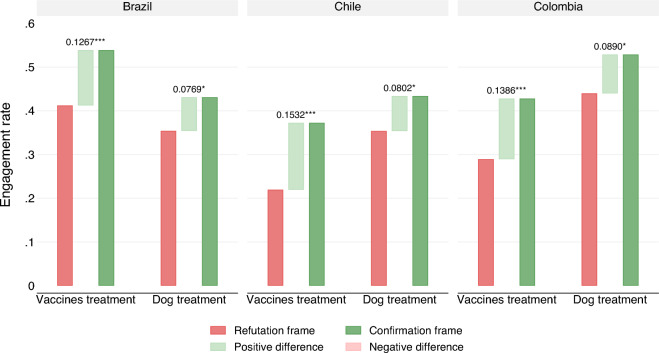
“Engagement” rate using the confirmation and refutation frames, TRUE or FALSE alternatively. Separate means are presented for the dog treatment (pseudo-placebo) and the vaccine treatments (pooling labeled and unlabeled treatments). The TRUE and FALSE statements are semantically identical but differ in their cognitive accessibility and their valence charge. Both the TRUE and FALSE adjudications are factually correct.

This treatment offers an opportunity to investigate the direct and unconditioned impact of the words “True” and “False”. Figure 7 includes the estimates of the “dog” treatment for Brazil, Chile, and Colombia. Full results are presented in Table S29 through Table S31 of the Supplemental Information File. Results point to positive and statistically significant effects of the confirmation frame on “engagement.” While these differences are smaller in magnitude to those of the vaccine treatments, they range between 8 and 9 percentage points and are statistically significant at the $p<.05$ level in Chile and Colombia. This simple exercise shows the effect of “TRUE” and “FALSE” frames unrelated to vaccines.

### A rejection of the cognitive difficulty hypothesis, H2

Our findings provide no evidence to suggest a higher cognitive burden associated with the FALSE frame, as hypothesized in Hypothesis 2 (H2). Two major observations support this conclusion. First, there is no consistent relationship between education level and engagement with the confirmation and refutation frames, indicated by the absence of significant patterns across countries. This can be seen in Table S15 of the Supplemental Information File (SIF). The influence of the confirmation frame across varying education levels, both on the propensity to react (Table S23) and the self-reported emotion (Table S24), does not show that more educated individuals are less susceptible to the frames.

**Figure 8:**
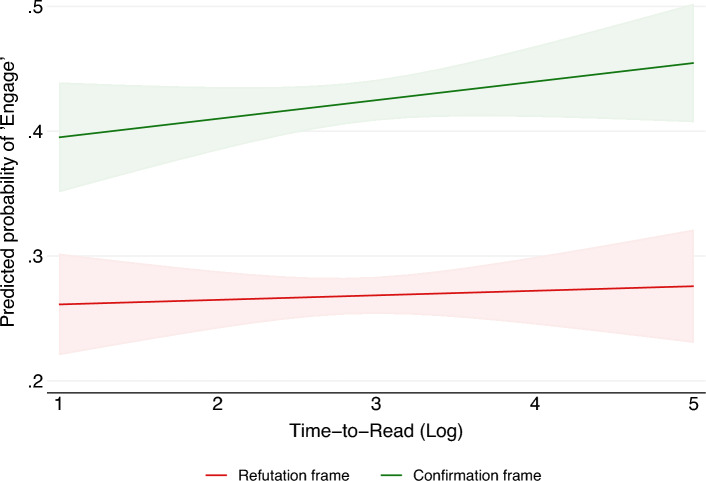
“Engagement” rate and Time-to-Read the Facebook Post in the vaccines treatments. Longer reading times are associated with larger differences in the response to the confirmation and refutation frames. The results refute the cognitive difficulty hypothesis, as greater attention does not reduce the differences between the *confirmation* and *refutation* frames. Probability estimates are obtained from a linear probability model, controlling for socio-demographic characteristics. The shaded area corresponds to the 95% confidence interval.

Second, we notice no significant decrease in the impact of the confirmation versus refutation frames attributable to the time respondents spent reading the treatments. Contrary to our expectations, longer reading times did not lessen the behavioral and emotional differences between the confirmation and refutation frames. In fact, in Argentina and Brazil, reading time correlates with a statistically significant increase in “likes” after exposure to the confirmation frame ($p<0.05$). The influence of reading time on overall engagement is shown in Fig. 8 for respondents from all countries and vaccine treatments.

This impact of extended exposure time is significant: prolonged exposure to the TRUE frame amplifies the differences in “likes” between the confirmation and refutation frames. Thus, a more thorough reading of the post increases the probability that the confirmation frame will attract a higher “like” rate than the refutation frame. Similar results are reported in Tables S25 and S26 of the SIF for all countries. Brazil demonstrates results analogous to Argentina’s, while Chile and Colombia show more modest positive correlations. We did not detect a statistically significant decline in engagement between the confirmation and refutation frames in any of the four countries. Consequently, the increased propensity to share the confirmation frame can be exclusively attributed to its positive valence charge, as per Hypothesis 3 (H3), rather than the cognitive difficulty associated with the refutation frame, as per Hypothesis 2 (H2).

### Other results: partisanship, vaccination status, and other sources of heterogeneity

In addition to heterogeneity in education and reading time, the Supplementary Information File presents additional exercises where we look at differences according to political affiliation, vaccination status, and other socio-demographic indicators. The differences in engagement remain for individuals in these different group categories. Confirmation frames about the vaccines tend to elicit more relative engagement and positive emotions among government supporters in Argentina, where the incumbent government aggressively pursued quarantine and mask mandates. On the other hand, the opposite is true for Brazil and Chile, where anti-COVID policies were more divisive and weakly enforced (see Tables S17, S18, S19, and S20 in the SIF).

Across the four countries, the differences in engagement and positive emotions tend to be higher for those who were vaccinated (twice or more) than those who were not (see Table S21 and S22 in the SIF). These results suggest that while the effects appear fairly universal, variations exist among different groups, in line with expectations. Therefore, the impact of different fact-checking strategies will not be uniform across all individuals. This result indicates that tailoring the framing and the message to suit specific demographics could still be desirable for maximizing the efficacy of the message.

## Discussion and limitations

The results of the four survey experiments support a higher intention to “engage” and “like” fact-checks framed as confirmations compared to the semantically equivalent refutation [Hypothesis 1 (H1)]. All four surveys provide evidence that the statistically significant findings remain robust across a variety of experimental designs, including different brands of the COVID-19 vaccine, with or without the use of labels, and across a diverse range of socio-demographic categories.

Moreover, the observed emotional responses and the absence of an effect on cognitive effort suggest that this discrepancy arises from distinct interpretations of the confirmation and refutation frames [Hypothesis 3 (H3)]. We speculate that, despite their semantic equivalence, confirmation frames draw the reader’s attention towards the health benefits of the vaccine, while refutation frames draw attention to the misinformation event itself.

The rejection of the cognitive burden hypothesis [Hypothesis 2 (H2)] further bolsters a valence-driven interpretation of the results. We find no evidence suggesting that rates of liking or sharing stem from difficulties in comprehending the confirmation and refutation frames. Nor is there a significant difference in the mean processing time for each frame. Intriguingly, we observe an increase in “likes” and “shares” for the confirmation frame with prolonged reading times. Given that the reading time is similar for both the confirmation and refutation frames, yet longer reading times increase the probability of liking and sharing the confirmation frame, the only plausible explanation is that a deeper understanding enhances the positive valence of the confirmation frame.

The results of our experiments have significant policy implications. Fact-checkers aiming to expand their posts’ reach would likely benefit from more frequent use of the confirmation frame. Our analysis of TRUE versus FALSE frames usage among 22 fact-checkers in Latin America revealed that refutation frames are four times more likely to be used. Some fact-checkers exclusively use refutation frames, potentially reducing their corrections’ exposure and likely increasing negative valence content on social media.

The findings in this paper also indicate that the effect of confirmation and refutation frames operates independently of other demographic, partisan, and health-associated moderators of fact-check sharing. The often emphasized negative partisan effects of misinformation can overshadow the fact that negative and positive valence charges in health messages are not solely a result of our partisan predispositions. Fact-checkers can choose different editorial strategies to frame a correction either as a contribution to the overall amount of correct information on social networks or the overall stock of polarized content. The standard use of the label “FALSE” can be seen not only as a warning about toxic content but also as a reminder to readers that social media is highly polarized. This decision may divert attention from crucial health issues and the underlying partisan conflict.

This study has some limitations that hopefully will be addressed in future experiments. As described in the methodology section, we only test the equivalent statements “It is TRUE that p” and “It is FALSE that not-p”. We did not test for the alternative equivalent statements, “It is TRUE that not-p” and “It is FALSE that p”. This was done explicitly, as we did not want to communicate false information to survey respondents, as would be the case if presented with the statements: “It is false that vaccines are effective” or “It is true that vaccines are not effective”. A limitation of this study is that the statement “It is FALSE that not-p” has two negative bits of information (“false” and “not effective”) compared to zero negative bits of information for the confirmation statement.

Similarly, a different set of experiments could be implemented using the equivalent statements “It is TRUE that p” and “It is FALSE that q,” where q is the antonym of p. The expected effect of using q instead of not-p should provide information to compare cases with one negative bit of information instead of two negative bits.

Finally, we provide convincing evidence that cognitive difficulty had no measurable effect in our vaccine experiment. However, we have not tested for this effect directly under varying levels of cognitive effort. Therefore, further tests would be required to measure whether engagement and sharing are insensitive to cognitive difficulty in larger experiments.

### Supplementary Information

Supplementary Information.

## Data Availability

All data generated for or used within this manuscript have been deposited at Harvard’s Dataverse and are publicly available here: https://doi.org/10.7910/DVN/UU4RUP.

## Code availability

All code for data analysis to generate the results in this manuscript have been deposited Harvard’s Dataverse and are publicly available here: https://doi.org/10.7910/DVN/UU4RUP.
