# Supplementary material for: Framing fact-checks as a “confirmation” increases engagement with corrections of misinformation: a four-country study
Source: Sci Rep. 2024 Feb 8;14:3201. doi: 10.1038/s41598-024-53337-0 (PMC10853194; doi:10.1038/s41598-024-53337-0)
Supplement: Supplementary file 1 — Supplementary Information. [file 41598_2024_53337_MOESM1_ESM.pdf]

Framing Fact-Checks as a "confirmation" increases engagement with corrections of misinformation: a four-country study.

Supplementary information

Natalia Aruguete* Flavia Batista † Ernesto Calvo ‡ Matias Guizzo Altube §

Carlos Scartascini ¶ Tiago Ventura ‖

January 22, 2024

This Supplemental Information File (SIF) complements the material presented in the main document. It includes the treatment designs, balance tables for all covariates and the treatment and control groups, the full regression models testing the hypotheses in the main document and the pre-registered experimental plan, and heterogeneous effects models conditioning on socio-demographic, partisan, and attention controls.

*Universidad Nacional de Quilmes, UNQ. Email: nataliaaruguete@gmail.com. Webpage: http://unq.academia.edu/nataliaaruguete.

†University of Maryland, Government and Politics, UMD. Address: 4118 Chiconteague, College Park, MD 20742, USA. Email: fbatista@umd.edu.

‡University of Maryland, Government and Politics, UMD. Address: 3140 Tydings Hall, College Park, MD 20742, USA. Email: ecalvo@umd.edu. Webpage: http://gvptsites.umd.edu/calvo/

§IADB. 1300 New York Avenue, N.W., Washington, DC 20577, USA. MATIASGU@iadb.org .

¶IADB. 1300 New York Avenue, N.W., Washington, DC 20577, USA. CARLOSSC@iadb.org.

‖University of Maryland, Government and Politics, UMD. Address: 4118 Chiconteague, College Park, MD 20742, USA. Email: venturat@umd.edu. Webpage: http://tiagoventura.rbind.io/

Contents

1 Treatment designs **3**

2 Balance tests for all specifications **8**

3 Differences of means in the general specification **16**

4 Differences of means in secondary specifications **21**

5 Heterogeneity in results **29**

5.1 Partisan attachment and vaccination status 33

5.2 Education and attention 44

6 Placebo experiment **54**

7 IRB Approval **59**

1 Treatment designs

As explained in the main document, respondents were exposed to a fictional Facebook post consisting of an image and a brief text. The text was either a confirmation of a correct statement or a refutation of an incorrect one. The main treatment communicates that vaccines are effective against Omicron in both the confirmation and refutation frames, while the placebo experiment reads that dogs do not understand human conversations. Variants of the main treatment consist in a rotation of the vaccine brand mentioned (being AstraZeneca, Moderna, and Sputnik V) in the Argentine experiment (see Figure S1) and the inclusion or not of a 'TRUE' or 'FALSE' label printed over the post image (a common practice in real fact-checking posts) in the Brazilian, Chilean, and Colombian experiments (see Figures S2 through S4). For details on the placebo experiment, refer to Section 6 of this document.

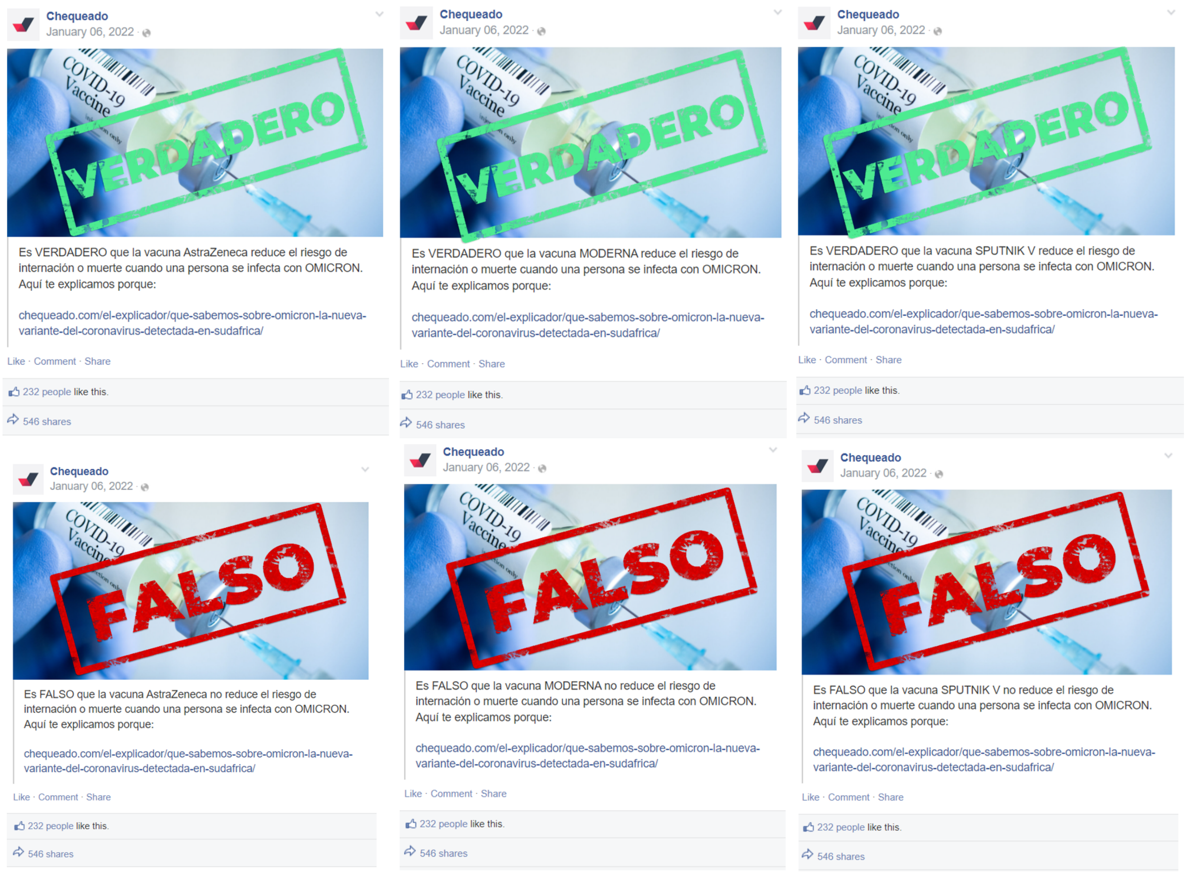

Figure S1 Images of the *confirmation* and *refutation* treatments used in Argentina. The confirmation and refutation frames are semantically equivalent but differ in their cognitive accessibility and their valence charge. The texts read "It is [TRUE/FALSE] that the [AstraZeneca/Moderna/Sputnik V] vaccine [reduces/does not reduce] the risk of hospitalization or death when a person gets infected with OMICRON". All six treatments are factually correct and conform to the design used by our partner organization in Argentina, *Chequeado*.

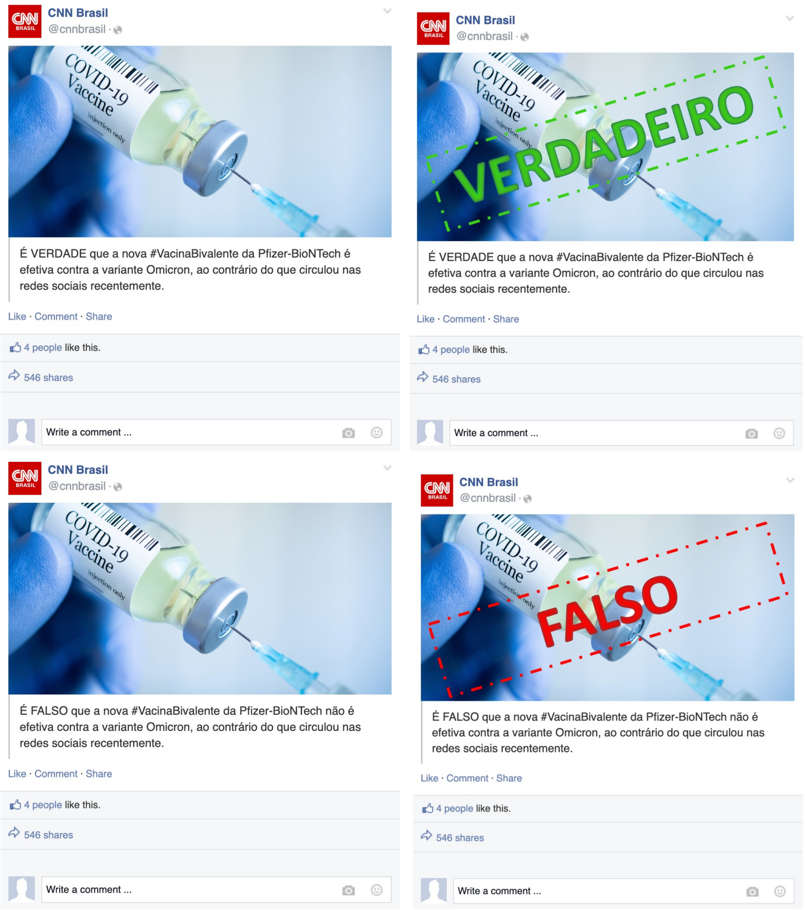

Figure S2 Images of the *confirmation* and *refutation* vaccine treatments used in Brazil. The confirmation and refutation frames are semantically equivalent but differ in their cognitive accessibility and their valence charge. The texts read "It is [TRUE/FALSE] that Pfizer-BioNTech's new #BivalentVaccine [is/is not] effective against the Omicron variant, contrary to what has been circulated on social media recently". Treatments on the left do not include a printed label and treatments on the right do include them. All four treatments are factually correct and conform to the design used by our partner organization in Argentina, *Chequeado*.

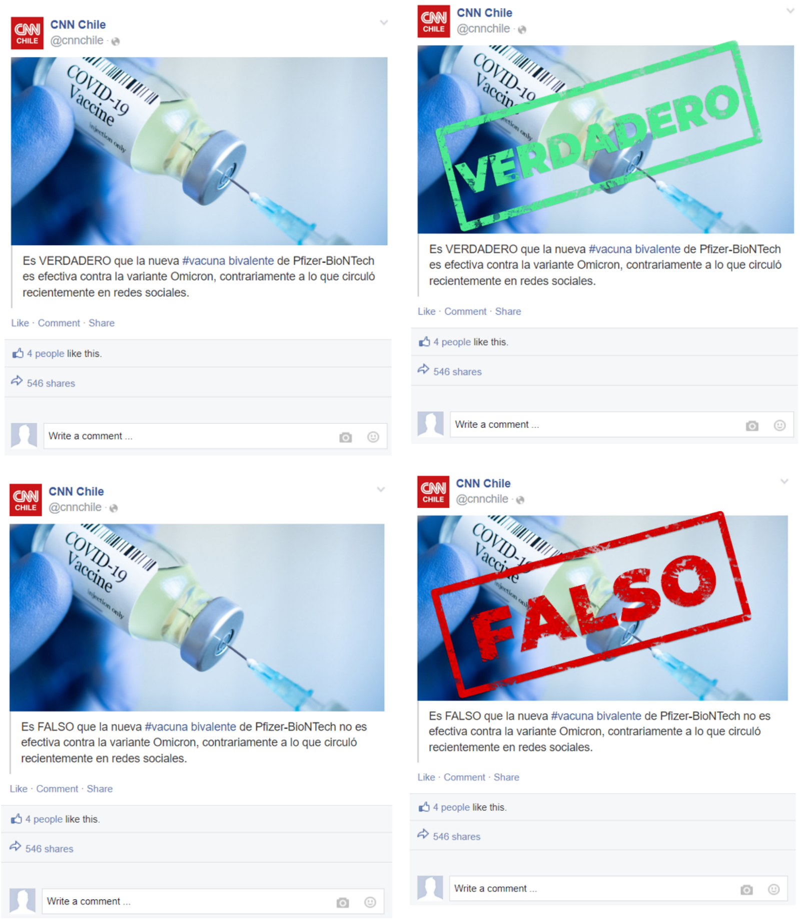

Figure S3 Images of the *confirmation* and *refutation* vaccine treatments used in Chile. The confirmation and refutation frames are semantically equivalent but differ in their cognitive accessibility and their valence charge. The texts read "It is [TRUE/FALSE] that Pfizer-BioNTech's new #BivalentVaccine [is/is not] effective against the Omicron variant, contrary to what has been circulated on social media recently". Treatments on the left do not include a printed label and treatments on the right do include them. All four treatments are factually correct and conform to the design used by our partner organization in Argentina, *Chequeado*.

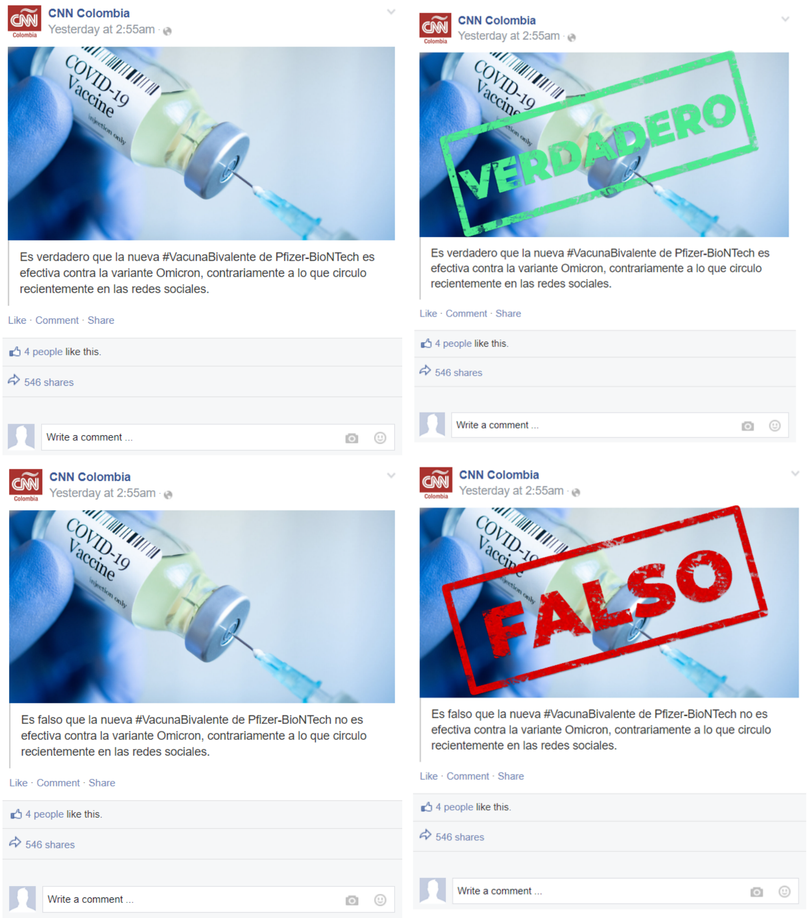

Figure S4 Images of the *confirmation* and *refutation* vaccine treatments used in Colombia. The confirmation and refutation frames are semantically equivalent but differ in their cognitive accessibility and their valence charge. The texts read "It is [TRUE/FALSE] that Pfizer-BioNTech's new #BivalentVaccine [is/is not] effective against the Omicron variant, contrary to what has been circulated on social media recently". Treatments on the left do not include a printed label and treatments on the right do include them. All four treatments are factually correct and conform to the design used by our partner organization in Argentina, *Chequeado*.

2 Balance tests for all specifications

Tables S1 to S4 describe the differences in sample means for key socio-demographic and partisan indicators used in this SIF file. Differences between average values of each control variable are computed for the confirmation framing versus refutation framing groups for the general specification, the specifications with and without printed 'TRUE' and 'FALSE' labels, the specifications with different vaccine brands, and the placebo experiment, respectively. Except for age (measured in years) and the logarithm of time spent reading, all variables are indicators and the differences presented are in percentage points.

Table S1 Balance tests for control variables in the general specification by country. Difference of means of selected control variables by confirmation and refutation frame assignment.

Variable	Refutation frame	Confirmation frame	Difference
Argentina			
Age (years)	40.66 (0.37)	40.29 (0.37)	-0.37 (0.52)
Educational attainment			
Incomplete secondary (or -)	6.34% (0.72)	5.18% (0.64)	-1.17% (0.96)
Completed secondary	19.03% (1.16)	20.28% (1.16)	1.26% (1.64)
Incomplete college	32.06% (1.38)	32.22% (1.35)	0.16% (1.93)
Completed college	35.01% (1.41)	35.89% (1.39)	0.88% (1.98)
Incomplete graduate (or +)	7.56% (0.78)	6.43% (0.71)	-1.13% (1.05)
Woman	54.91% (1.47)	57.26% (1.43)	2.35% (2.05)
Employed	79.93% (1.18)	80.05% (1.16)	0.12% (1.65)
Vote for incumbent party	27.28% (1.31)	24.21% (1.24)	-3.07% (1.8)
Vote for opposition party	43.96% (1.46)	44.91% (1.44)	0.95% (2.05)
Time to read (log of seconds)	2.84(0.02)	2.78(0.02)	-0.06(0.03)
Have had COVID-19	43.53% (1.46)	44.16% (1.44)	0.63% (2.05)
Vaccines against COVID-19			
Non-vaccinated against COVID-19	5.85% (0.69)	6.19% (0.7)	0.35% (0.98)
Vaccinated once	2.88% (0.49)	3.35% (0.52)	0.47% (0.72)
Vaccinated twice (or +)	91.27% (0.83)	90.46% (0.85)	-0.81% (1.19)
Brazil			
Age (years)	39.44 (0.42)	39.96 (0.41)	0.52 (0.59)
Educational attainment			
Incomplete secondary (or -)	9.7% (1.03)	8.62% (0.98)	-1.08% (1.42)
Completed secondary	28.12% (1.57)	30.95% (1.61)	2.83% (2.25)
Incomplete college	14.3% (1.22)	12.74% (1.16)	-1.56% (1.68)
Completed college	26.42% (1.54)	27.55% (1.56)	1.12% (2.19)
Incomplete graduate (or +)	21.45% (1.43)	20.15% (1.4)	-1.31% (2)
Woman	49.58% (1.74)	47.57% (1.74)	-2% (2.46)
Employed	77.16% (1.46)	77.4% (1.46)	0.24% (2.07)
Vote for incumbent party	38.42% (1.69)	38.59% (1.7)	0.17% (2.4)
Vote for opposition party	42.06% (1.72)	43.08% (1.73)	1.02% (2.44)
Time to read (log of seconds)	2.79(0.03)	2.82(0.03)	0.03(0.04)
Have had COVID-19	45.07% (1.74)	47.98% (1.75)	2.91% (2.47)
Vaccines against COVID-19			
Non-vaccinated against COVID-19	5.71% (0.81)	4.88% (0.75)	-0.83% (1.11)
Vaccinated once	4.25% (0.7)	3.29% (0.62)	-0.96% (0.94)
Vaccinated twice (or +)	90.04% (1.04)	91.83% (0.96)	1.79% (1.42)
Chile			
Age (years)	42.13 (0.43)	41.8 (0.44)	-0.33 (0.62)
Educational attainment			

Incomplete secondary (or -)	1.48% (0.42)	1.97% (0.49)	0.49% (0.65)
Completed secondary	20.47% (1.42)	20.91% (1.43)	0.44% (2.01)
Incomplete college	20.72% (1.42)	21.16% (1.43)	0.44% (2.02)
Completed college	46.61% (1.75)	44.16% (1.74)	-2.45% (2.47)
Incomplete graduate (or +)	10.73% (1.09)	11.81% (1.13)	1.08% (1.57)
Woman	59.8% (1.72)	57.69% (1.73)	-2.12% (2.44)
Employed	77.01% (1.48)	79.43% (1.42)	2.42% (2.05)
Vote for incumbent party	43.03% (1.74)	43.79% (1.74)	0.76% (2.46)
Vote for opposition party	35.76% (1.68)	32.35% (1.64)	-3.41% (2.35)
Time to read (log of seconds)	2.92 (0.02)	3.06 (0.03)	0.13*** (0.04)
Have had COVID-19	39.85% (1.72)	40.27% (1.72)	0.42% (2.44)
Vaccines against COVID-19			
Non-vaccinated against COVID-19	3.58% (0.65)	4.07% (0.69)	0.49% (0.95)
Vaccinated once	1.23% (0.39)	1.36% (0.41)	0.12% (0.56)
Vaccinated twice (or +)	95.19% (0.75)	94.57% (0.8)	-0.61% (1.1)
Colombia			
Age (years)	37.81 (0.42)	37.42 (0.45)	-0.39 (0.62)
Educational attainment			
Incomplete secondary (or -)	2.73% (0.58)	3.47% (0.67)	0.73% (0.88)
Completed secondary	22.86% (1.48)	22.27% (1.52)	-0.59% (2.12)
Incomplete college	23.6% (1.5)	23.6% (1.55)	0% (2.16)
Completed college	40.12% (1.73)	40.4% (1.79)	0.28% (2.49)
Incomplete graduate (or +)	10.68% (1.09)	10.27% (1.11)	-0.42% (1.55)
Woman	52.17% (1.76)	54.93% (1.82)	2.76% (2.53)
Employed	80.87% (1.39)	84.11% (1.34)	3.24% (1.93)
Vote for incumbent party	51.18% (1.76)	47.2% (1.82)	-3.98% (2.54)
Vote for opposition party	20.12% (1.41)	18.13% (1.41)	-1.99% (2)
Time to read (log of seconds)	2.97 (0.03)	3.07 (0.03)	0.09* (0.04)
Have had COVID-19	41.32% (1.74)	42.7% (1.81)	1.38% (2.51)
Vaccines against COVID-19			
Non-vaccinated against COVID-19	5.35% (0.79)	7.1% (0.94)	1.74% (1.23)
Vaccinated once	10.09% (1.06)	10.98% (1.14)	0.89% (1.56)
Vaccinated twice (or +)	84.56% (1.28)	81.93% (1.41)	-2.63% (1.9)

Note: Robust standard errors in parentheses. *** $p < 0.001$, ** $p < 0.01$, * $p < 0.05$. The first and second columns represent the average value of each control variable for the refutation frame and confirmation frame groups respectively. Significance levels of differences presented in the third column are derived from linear regression models without controls.

Table S2 Balance tests for control variables in the Brazil, Chile, and Colombia surveys by use of labels. Difference of means of selected control variables by confirmation and refutation frame assignment for the specifications with and without printed 'TRUE' and 'FALSE' labels.

	With labels			Without labels		
Variable	Refutation	Confirmation	Difference	Refutation	Confirmation	Difference
Brazil						
Age (years)	39.13 (0.59)	40.04 (0.59)	0.91 (0.84)	39.76 (0.59)	39.9 (0.58)	0.14 (0.83)
Educational attainment						
Incomplete secondary (or -)	10.6% (1.51)	9.25% (1.47)	-1.35 (2.11)	8.78% (1.4)	8.05% (1.31)	-0.73 (1.91)
Completed secondary	27.95% (2.21)	30.08% (2.33)	2.13 (3.21)	28.29% (2.23)	31.72% (2.23)	3.43 (3.15)
Incomplete college	14.7% (1.74)	12.85% (1.7)	-1.85 (2.43)	13.9% (1.71)	12.64% (1.6)	-1.26 (2.34)
Completed college	26.02% (2.16)	25.96% (2.23)	-0.06 (3.1)	26.83% (2.19)	28.97% (2.18)	2.14 (3.09)
Incomplete graduate (or +)	20.72% (1.99)	21.85% (2.1)	1.13 (2.89)	22.2% (2.05)	18.62% (1.87)	-3.57 (2.78)
Woman	51.08% (2.46)	48.59% (2.54)	-2.5 (3.53)	48.05% (2.47)	46.67% (2.39)	-1.38 (3.44)
Employed	77.97% (2.04)	76.8% (2.15)	-1.16 (2.96)	76.34% (2.1)	77.93% (1.99)	1.59 (2.89)
Vote for incumbent party	38.07% (2.39)	41.39% (2.5)	3.32 (3.46)	38.78% (2.41)	36.09% (2.31)	-2.69 (3.33)
Vote for opposition party	41.69% (2.42)	39.85% (2.49)	-1.84 (3.47)	42.44% (2.44)	45.98% (2.39)	3.54 (3.42)
Time to read (log of seconds)	2.8 (0.04)	2.86 (0.04)	0.06 (0.05)	2.79 (0.04)	2.8 (0.04)	0.01 (0.05)
Have had COVID-19	44.77% (2.46)	48.84% (2.54)	4.07 (3.54)	45.37% (2.46)	47.21% (2.41)	1.84 (3.45)
Vaccines against COVID-19						
Non-vaccinated against COVID-19	5.57% (1.13)	6.96% (1.29)	1.39 (1.72)	5.85% (1.16)	3.01% (0.82)	-2.84* (1.42)
Vaccinated once	3.39% (0.89)	3.35% (0.91)	-0.04 (1.28)	5.12% (1.09)	3.24% (0.85)	-1.88 (1.38)
Vaccinated twice (or +)	91.04% (1.41)	89.69% (1.55)	-1.35 (2.09)	89.02% (1.55)	93.75% (1.17)	4.73* (1.94)
Chile						
Age (years)	41.05 (0.63)	42.07 (0.62)	1.03 (0.88)	43.15 (0.6)	41.53 (0.61)	-1.62 (0.86)
Educational attainment						
Incomplete secondary (or -)	1.53% (0.62)	1.99% (0.7)	0.46 (0.93)	1.44% (0.58)	1.95% (0.68)	0.51 (0.9)
Completed secondary	20.87% (2.05)	20.4% (2.01)	-0.47 (2.87)	20.1% (1.96)	21.41% (2.03)	1.32 (2.82)
Incomplete college	21.63% (2.08)	20.15% (2)	-1.48 (2.89)	19.86% (1.95)	22.14% (2.05)	2.28 (2.83)
Completed college	45.55% (2.52)	46.77% (2.49)	1.22 (3.54)	47.61% (2.45)	41.61% (2.43)	-6 (3.45)
Incomplete graduate (or +)	10.43% (1.54)	10.7% (1.54)	0.26 (2.18)	11% (1.53)	12.9% (1.66)	1.89 (2.26)
Woman	58.27% (2.49)	56.97% (2.47)	-1.3 (3.51)	61.24% (2.39)	58.39% (2.43)	-2.85 (3.41)

Employed	76.21% (2.16)	82.29% (1.91)	6.08* (2.88)	77.75% (2.04)	76.64% (2.09)	-1.11 (2.92)
Vote for incumbent party	41.98% (2.49)	43.78% (2.48)	1.8 (3.51)	44.02% (2.43)	43.8% (2.45)	-0.22 (3.45)
Vote for opposition party	35.88% (2.42)	34.33% (2.37)	-1.55 (3.39)	35.65% (2.35)	30.41% (2.27)	-5.23 (3.27)
Time to read (log of seconds)	2.87 (0.03)	3.09 (0.04)	0.22*** (0.05)	2.98 (0.03)	3.03 (0.04)	0.05 (0.05)
Have had COVID-19	38.01% (2.45)	41.15% (2.46)	3.14 (3.48)	41.59% (2.42)	39.42% (2.41)	-2.17 (3.42)
Vaccines against COVID-19						
Non-vaccinated against COVID-19	4.33% (1.03)	4.24% (1.01)	-0.09 (1.44)	2.88% (0.82)	3.9% (0.96)	1.02 (1.26)
Vaccinated once	1.27% (0.57)	1.25% (0.55)	-0.03 (0.79)	1.2% (0.53)	1.46% (0.59)	0.26 (0.8)
Vaccinated twice (or +)	94.4% (1.16)	94.51% (1.14)	0.11 (1.63)	95.92% (0.97)	94.63% (1.11)	-1.29 (1.48)
Colombia						
Age (years)	38.34 (0.61)	38.01 (0.67)	-0.33 (0.9)	37.3 (0.57)	36.85 (0.61)	-0.46 (0.84)
Educational attainment						
Incomplete secondary (or -)	2.53% (0.79)	4.02% (1.02)	1.49 (1.29)	2.93% (0.83)	2.92% (0.87)	-0.01 (1.2)
Completed secondary	22.53% (2.1)	25.2% (2.25)	2.67 (3.08)	23.17% (2.09)	19.36% (2.04)	-3.81 (2.92)
Incomplete college	22.53% (2.1)	21.45% (2.13)	-1.08 (2.99)	24.63% (2.13)	25.73% (2.25)	1.1 (3.1)
Completed college	40% (2.47)	40.21% (2.54)	0.21 (3.54)	40.24% (2.42)	40.58% (2.53)	0.34 (3.51)
Incomplete graduate (or +)	12.41% (1.66)	9.12% (1.49)	-3.29 (2.23)	9.02% (1.42)	11.41% (1.64)	2.38 (2.17)
Woman	48.1% (2.52)	52.55% (2.59)	4.45 (3.61)	56.1% (2.45)	57.29% (2.55)	1.2 (3.54)
Employed	82.03% (1.93)	84.45% (1.88)	2.43 (2.7)	79.76% (1.99)	83.78% (1.9)	4.02 (2.75)
Vote for incumbent party	52.15% (2.52)	45.84% (2.58)	-6.31 (3.61)	50.24% (2.47)	48.54% (2.58)	-1.7 (3.57)
Vote for opposition party	22.03% (2.09)	17.43% (1.97)	-4.6 (2.87)	18.29% (1.91)	18.83% (2.02)	0.54 (2.78)
Time to read (log of seconds)	2.98 (0.04)	3.09 (0.04)	0.11 (0.06)	2.96 (0.04)	3.04 (0.04)	0.08 (0.05)
Have had COVID-19	41.98% (2.49)	40.97% (2.56)	-1.01 (3.57)	40.69% (2.44)	44.41% (2.57)	3.73 (3.54)
Vaccines against COVID-19						
Non-vaccinated against COVID-19	6.35% (1.23)	7.28% (1.35)	0.93 (1.83)	4.4% (1.02)	6.91% (1.31)	2.51 (1.66)
Vaccinated once	10.66% (1.56)	10.78% (1.61)	0.12 (2.24)	9.54% (1.45)	11.17% (1.63)	1.63 (2.18)
Vaccinated twice (or +)	82.99% (1.9)	81.94% (2)	-1.05 (2.76)	86.06% (1.71)	81.91% (1.99)	-4.15 (2.62)

Note: Robust standard errors in parentheses. *** $p < 0.001$, ** $p < 0.01$, * $p < 0.05$. Columns labeled as Refutation and Confirmation present the average value of each control variable for the refutation frame and confirmation frame groups respectively, either in the treatment branch that includes visual labels of TRUE and FALSE or in the treatment branch that does not include the labels. Significance levels of the differences presented are derived from linear regression models without controls.

Table S3 Balance tests for control variables in the Argentina survey by mentioned vaccine brand. Difference of means of selected control variables by confirmation and refutation frame assignment for each brand of vaccines mentioned in the vignette in the Argentina survey.

Variable	Refutation frame	Confirmation frame	Difference
Moderna			
Age (years)	40.89 (0.66)	40.18 (0.63)	-0.71 (0.91)
Educational attainment			
Incomplete secondary (or -)	6.55% (1.24)	5.76% (1.17)	-0.78 (1.71)
Completed secondary	19.14% (1.98)	19.8% (2)	0.66 (2.81)
Incomplete college	32.49% (2.35)	31.83% (2.33)	-0.66 (3.32)
Completed college	34.26% (2.38)	36.34% (2.41)	2.08 (3.39)
Incomplete graduate (or +)	7.56% (1.33)	6.27% (1.21)	-1.29 (1.8)
Woman	54.41% (2.5)	56.89% (2.48)	2.48 (3.53)
Employed	77.78% (2.09)	81.7% (1.94)	3.93 (2.85)
Vote for incumbent party	26.7% (2.22)	22.81% (2.1)	-3.89 (3.06)
Vote for opposition party	46.35% (2.51)	46.37% (2.5)	0.02 (3.54)
Time to read (log of seconds)	2.87 (0.04)	2.79 (0.04)	-0.07 (0.06)
Have had COVID-19	44.08% (2.49)	44.61% (2.49)	0.53 (3.53)
Vaccines against COVID-19			
Non-vaccinated against COVID-19	4.8% (1.08)	6.55% (1.24)	1.75 (1.64)
Vaccinated once	2.78% (0.83)	3.78% (0.96)	1 (1.27)
Vaccinated twice (or +)	92.42% (1.33)	89.67% (1.53)	-2.75 (2.03)
Sputnik			
Age (years)	40.19 (0.66)	40.28 (0.62)	0.08 (0.9)
Educational attainment			
Incomplete secondary (or -)	6.5% (1.31)	4.52% (1.04)	-1.97 (1.68)
Completed secondary	15.82% (1.94)	22.11% (2.08)	6.29* (2.85)
Incomplete college	34.18% (2.52)	31.41% (2.33)	-2.77 (3.44)
Completed college	36.44% (2.56)	36.43% (2.42)	-0.01 (3.52)
Incomplete graduate (or +)	7.06% (1.36)	5.53% (1.15)	-1.53 (1.78)
Woman	53.95% (2.65)	55.78% (2.49)	1.82 (3.64)
Employed	80.68% (2.11)	78.89% (2.05)	-1.79 (2.94)
Vote for incumbent party	26.84% (2.36)	26.38% (2.21)	-0.45 (3.23)
Vote for opposition party	46.05% (2.65)	41.71% (2.47)	-4.34 (3.63)
Time to read (log of seconds)	2.78 (0.04)	2.74 (0.04)	-0.04 (0.06)
Have had COVID-19	45.48% (2.65)	43.47% (2.49)	-2.01 (3.64)
Vaccines against COVID-19			
Non-vaccinated against COVID-19	7.43% (1.4)	6.03% (1.19)	-1.4 (1.84)
Vaccinated once	2.57% (0.85)	3.77% (0.96)	1.2 (1.28)
Vaccinated twice (or +)	90% (1.61)	90.2% (1.49)	0.2 (2.19)
AstraZeneca			
Age (years)	40.85 (0.6)	40.42 (0.66)	-0.43 (0.89)

Educational attainment			
Incomplete secondary (or -)	6% (1.19)	5.24% (1.11)	-0.76 (1.63)
Completed secondary	21.75% (2.07)	18.95% (1.96)	-2.8 (2.85)
Incomplete college	29.75% (2.29)	33.42% (2.36)	3.67 (3.29)
Completed college	34.5% (2.38)	34.91% (2.38)	0.41 (3.37)
Incomplete graduate (or +)	8% (1.36)	7.48% (1.32)	-0.52 (1.89)
Woman	56.25% (2.48)	59.1% (2.46)	2.85 (3.49)
Employed	81.41% (1.95)	79.55% (2.02)	-1.86 (2.81)
Vote for incumbent party	28.25% (2.25)	23.44% (2.12)	-4.81 (3.09)
Vote for opposition party	39.75% (2.45)	46.63% (2.49)	6.88* (3.5)
Time to read (log of seconds)	2.86 (0.04)	2.8 (0.04)	-0.06 (0.05)
Have had COVID-19	41.25% (2.46)	44.39% (2.48)	3.14 (3.5)
Vaccines against COVID-19			
Non-vaccinated against COVID-19	5.5% (1.14)	6% (1.19)	0.5 (1.65)
Vaccinated once	3.25% (0.89)	2.5% (0.78)	-0.75 (1.18)
Vaccinated twice (or +)	91.25% (1.41)	91.5% (1.4)	0.25 (1.99)

Note: Robust standard errors in parentheses. *** p < 0.001, ** p < 0.01, * p < 0.05. The first and second columns represent the average value of each control variable for the refutation frame and confirmation frame groups respectively. Significance levels of differences presented in the third column are derived from linear regression models without controls.

Table S4 Balance tests for control variables in the placebo experiment by country. Difference of means of selected control variables by confirmation and refutation frame assignment.

Variable	Refutation frame	Confirmation frame	Difference
Brazil			
Age (years)	39.3 (0.63)	39.52 (0.63)	0.22 (0.89)
Educational attainment			
Incomplete secondary (or -)	7.36% (1.36)	10.39% (1.56)	3.03 (2.07)
Completed secondary	25.89% (2.29)	26.75% (2.26)	0.87 (3.22)
Incomplete college	18.53% (2.03)	16.62% (1.9)	-1.91 (2.78)
Completed college	29.97% (2.39)	26.75% (2.26)	-3.22 (3.29)
Incomplete graduate (or +)	18.26% (2.02)	19.48% (2.02)	1.22 (2.86)
Woman	50.14% (2.61)	52.99% (2.55)	2.85 (3.65)
Employed	74.73% (2.28)	70.65% (2.32)	-4.08 (3.26)
Vote for incumbent party	40.05% (2.56)	34.81% (2.43)	-5.25 (3.53)
Vote for opposition party	40.33% (2.56)	45.45% (2.54)	5.13 (3.61)
Time to read (log of seconds)	2.69 (0.04)	2.69 (0.03)	0 (0.05)
Have had COVID-19	41.69% (2.58)	41.3% (2.51)	-0.39 (3.6)
Vaccines against COVID-19			
Non-vaccinated against COVID-19	5.18% (1.16)	4.42% (1.05)	-0.76 (1.56)
Vaccinated once	2.72% (0.85)	3.64% (0.96)	0.91 (1.28)

Vaccinated twice (or +)	92.1% (1.41)	91.95% (1.39)	-0.15 (1.98)
Chile			
Age (years)	43.09 (0.66)	41.34 (0.62)	-1.76 (0.91)
Educational attainment			
Incomplete secondary (or -)	1.39% (0.62)	1.25% (0.55)	-0.15 (0.83)
Completed secondary	14.21% (1.85)	20.2% (2.01)	5.99* (2.73)
Incomplete college	23.96% (2.26)	21.7% (2.06)	-2.26 (3.06)
Completed college	49.58% (2.64)	47.63% (2.5)	-1.95 (3.64)
Incomplete graduate (or +)	10.86% (1.64)	9.23% (1.45)	-1.64 (2.19)
Woman	55.71% (2.63)	55.36% (2.49)	-0.35 (3.62)
Employed	81.62% (2.05)	81.2% (1.96)	-0.41 (2.83)
Vote for incumbent party	39.83% (2.59)	39.4% (2.44)	-0.43 (3.56)
Vote for opposition party	37.05% (2.55)	35.66% (2.39)	-1.39 (3.5)
Time to read (log of seconds)	2.97 (0.04)	2.86 (0.04)	-0.11* (0.05)
Have had COVID-19	39.39% (2.59)	40.65% (2.46)	1.26 (3.57)
Vaccines against COVID-19			
Non-vaccinated against COVID-19	6.15% (1.27)	5.74% (1.16)	-0.41 (1.72)
Vaccinated once	1.4% (0.62)	1.25% (0.55)	-0.15 (0.83)
Vaccinated twice (or +)	92.46% (1.4)	93.02% (1.27)	0.56 (1.89)
Colombia			
Age (years)	37.17 (0.6)	37.63 (0.59)	0.46 (0.85)
Educational attainment			
Incomplete secondary (or -)	2.7% (0.8)	2.88% (0.82)	0.18 (1.15)
Completed secondary	18.43% (1.92)	19.95% (1.96)	1.52 (2.75)
Incomplete college	25.31% (2.16)	22.84% (2.06)	-2.47 (2.98)
Completed college	41.77% (2.45)	41.35% (2.42)	-0.42 (3.44)
Incomplete graduate (or +)	11.79% (1.6)	12.98% (1.65)	1.19 (2.3)
Woman	53.07% (2.48)	54.81% (2.44)	1.74 (3.48)
Employed	81.08% (1.94)	82.69% (1.86)	1.61 (2.69)
Vote for incumbent party	49.14% (2.48)	47.36% (2.45)	-1.78 (3.49)
Vote for opposition party	18.18% (1.91)	15.14% (1.76)	-3.04 (2.6)
Time to read (log of seconds)	2.86 (0.04)	2.81 (0.03)	-0.06 (0.05)
Have had COVID-19	42.36% (2.46)	46.27% (2.45)	3.9 (3.47)
Vaccines against COVID-19			
Non-vaccinated against COVID-19	6.65% (1.24)	5.56% (1.13)	-1.09 (1.67)
Vaccinated once	8.62% (1.39)	9.18% (1.42)	0.56 (1.99)
Vaccinated twice (or +)	84.73% (1.79)	85.27% (1.74)	0.54 (2.5)

Note: Robust standard errors in parentheses. *** $p < 0.001$, ** $p < 0.01$, * $p < 0.05$. The first and second columns represent the average value of each control variable for the refutation frame and confirmation frame groups respectively in the placebo experiment. Significance levels of differences presented in the third column are derived from linear regression models without controls.

3 Differences of means in the general specification

Tables S5 to S8 expand the results from Table 1 of the main article. They present the detailed differences of means of the dependent variables (reactions and emotions generated) between the confirmation and refutation frames in the general specification for each specific country survey (Argentina, Brazil, Chile, and Colombia, respectively). Both frames are semantically equivalent propositions phrased as a confirmation of accurate information or as refutation of inaccurate information. The results in these tables can be used to compare findings in the complementary specifications (varying the vaccine brand, the use of printed 'TRUE' or 'FALSE' labels, heterogeneous specifications, and the placebo experiment).

Table S5 Difference of means between the confirmation and refutation frames in the Argentina survey.

Variable	Refutation frame	Confirmation frame	Simple difference	Difference with controls
Reactions				
Engage	0.189	0.371	0.182***	0.188***
	(0.012)	(0.014)	(0.018)	(0.018)
Like	0.084	0.245	0.160***	0.163***
	(0.008)	(0.012)	(0.015)	(0.015)
Share	0.076	0.115	0.039**	0.042***
	(0.008)	(0.009)	(0.012)	(0.012)
Comment	0.043	0.048	0.006	0.008
	(0.006)	(0.006)	(0.009)	(0.009)
Emotions				
Optimistic	0.060	0.333	0.273***	0.278***
	(0.007)	(0.014)	(0.015)	(0.015)
Joyful	0.017	0.098	0.082***	0.087***
	(0.004)	(0.009)	(0.009)	(0.009)
Angry	0.165	0.045	-0.120***	-0.121***
	(0.011)	(0.006)	(0.012)	(0.012)
Sad	0.058	0.023	-0.035***	-0.035***
	(0.007)	(0.004)	(0.008)	(0.008)
Stressed	0.128	0.058	-0.069***	-0.072***
	(0.010)	(0.007)	(0.012)	(0.012)
Fearful	0.043	0.028	-0.015	-0.015
	(0.006)	(0.005)	(0.008)	(0.008)
Disgusted	0.195	0.076	-0.119***	-0.121***
	(0.012)	(0.008)	(0.014)	(0.014)
Indifferent	0.443	0.417	-0.027	-0.028
	(0.015)	(0.014)	(0.020)	(0.020)

Note: Robust standard errors in parentheses. *** $p < 0.001$, ** $p < 0.01$, * $p < 0.05$. The first two columns represent average values for each reaction and emotion in response to the refutation and confirmation frames respectively. The third column presents the difference between the confirmation and refutation frame without controls. Differences in the fourth column are estimated controlling for age, sex, educational attainment, employment status, partisan attachment, having had COVID-19, number of doses administered of COVID-19 vaccine, and time spent reading the post. Significance levels are derived from linear regression models.

Table S6 Difference of means between the confirmation and refutation frames in the Brazil survey.

Variable	Refutation frame	Confirmation frame	Simple difference	Difference with controls
Reactions				
Engage	0.412	0.539	0.127***	0.131***
	(0.017)	(0.017)	(0.024)	(0.024)
Like	0.275	0.402	0.127***	0.127***
	(0.016)	(0.017)	(0.023)	(0.022)
Share	0.148	0.147	-0.001	0.001
	(0.012)	(0.012)	(0.017)	(0.018)
Comment	0.104	0.104	0.000	-0.002
	(0.011)	(0.011)	(0.015)	(0.015)
Emotions				
Optimistic	0.178	0.444	0.266***	0.258***
	(0.013)	(0.017)	(0.022)	(0.022)
Joyful	0.103	0.210	0.107***	0.106***
	(0.011)	(0.014)	(0.018)	(0.018)
Angry	0.125	0.040	-0.085***	-0.083***
	(0.012)	(0.007)	(0.013)	(0.014)
Sad	0.136	0.033	-0.103***	-0.102***
	(0.012)	(0.006)	(0.013)	(0.014)
Stressed	0.091	0.036	-0.055***	-0.051***
	(0.010)	(0.007)	(0.012)	(0.012)
Fearful	0.115	0.059	-0.056***	-0.051***
	(0.011)	(0.008)	(0.014)	(0.014)
Disgusted	0.147	0.038	-0.109***	-0.112***
	(0.012)	(0.007)	(0.014)	(0.014)
Indifferent	0.343	0.316	-0.027	-0.029
	(0.017)	(0.016)	(0.023)	(0.023)

Note: Robust standard errors in parentheses. *** $p < 0.001$, ** $p < 0.01$, * $p < 0.05$. The first two columns represent average values for each reaction and emotion in response to the refutation and confirmation frames respectively. The third column presents the difference between the confirmation and refutation frame without controls. Differences in the fourth column are estimated controlling for age, sex, educational attainment, employment status, partisan attachment, having had COVID-19, number of doses administered of COVID-19 vaccine, and time spent reading the post. Significance levels are derived from linear regression models.

Table S7 Difference of means between the confirmation and refutation frames in the Chile survey.

Variable	Refutation frame	Confirmation frame	Simple difference	Difference with controls
Reactions				
Engage	0.219	0.373	0.153***	0.152***
	(0.015)	(0.017)	(0.022)	(0.023)
Like	0.086	0.257	0.171***	0.171***
	(0.010)	(0.015)	(0.018)	(0.018)
Share	0.122	0.137	0.014	0.010
	(0.012)	(0.012)	(0.017)	(0.017)
Comment	0.039	0.048	0.009	0.006
	(0.007)	(0.007)	(0.010)	(0.010)
Emotions				
Optimistic	0.095	0.338	0.243***	0.246***
	(0.010)	(0.017)	(0.020)	(0.020)
Joyful	0.028	0.135	0.107***	0.105***
	(0.006)	(0.012)	(0.013)	(0.014)
Angry	0.144	0.062	-0.083***	-0.081***
	(0.012)	(0.008)	(0.015)	(0.015)
Sad	0.043	0.018	-0.025**	-0.023**
	(0.007)	(0.005)	(0.009)	(0.009)
Stressed	0.113	0.086	-0.027	-0.025
	(0.011)	(0.010)	(0.015)	(0.015)
Fearful	0.116	0.074	-0.042**	-0.042**
	(0.011)	(0.009)	(0.015)	(0.015)
Disgusted	0.137	0.091	-0.046**	-0.046**
	(0.012)	(0.010)	(0.016)	(0.015)
Indifferent	0.503	0.406	-0.097***	-0.102***
	(0.018)	(0.017)	(0.025)	(0.025)

Note: Robust standard errors in parentheses. *** $p < 0.001$, ** $p < 0.01$, * $p < 0.05$. The first two columns represent average values for each reaction and emotion in response to the refutation and confirmation frames respectively. The third column presents the difference between the confirmation and refutation frame without controls. Differences in the fourth column are estimated controlling for age, sex, educational attainment, employment status, partisan attachment, having had COVID-19, number of doses administered of COVID-19 vaccine, and time spent reading the post. Significance levels are derived from linear regression models.

Table S8 Difference of means between the confirmation and refutation frames in the Colombia survey.

Variable	Refutation frame	Confirmation frame	Simple difference	Difference with controls
Reactions				
Engage	0.289	0.428	0.139***	0.147***
	(0.016)	(0.018)	(0.024)	(0.024)
Like	0.124	0.240	0.116***	0.120***
	(0.012)	(0.016)	(0.019)	(0.020)
Share	0.152	0.184	0.032	0.035
	(0.013)	(0.014)	(0.019)	(0.019)
Comment	0.051	0.077	0.026*	0.026*
	(0.008)	(0.010)	(0.012)	(0.012)
Emotions				
Optimistic	0.142	0.363	0.221***	0.226***
	(0.012)	(0.018)	(0.021)	(0.022)
Joyful	0.032	0.101	0.069***	0.071***
	(0.006)	(0.011)	(0.013)	(0.013)
Angry	0.096	0.047	-0.049***	-0.049***
	(0.010)	(0.008)	(0.013)	(0.013)
Sad	0.078	0.032	-0.046***	-0.049***
	(0.009)	(0.006)	(0.011)	(0.011)
Stressed	0.108	0.080	-0.028	-0.028
	(0.011)	(0.010)	(0.015)	(0.015)
Fearful	0.173	0.140	-0.033	-0.037*
	(0.013)	(0.013)	(0.018)	(0.019)
Disgusted	0.051	0.051	-0.000	0.001
	(0.008)	(0.008)	(0.011)	(0.011)
Indifferent	0.516	0.381	-0.134***	-0.133***
	(0.018)	(0.018)	(0.025)	(0.025)

Note: Robust standard errors in parentheses. *** $p < 0.001$, ** $p < 0.01$, * $p < 0.05$. The first two columns represent average values for each reaction and emotion in response to the refutation and confirmation frames respectively. The third column presents the difference between the confirmation and refutation frame without controls. Differences in the fourth column are estimated controlling for age, sex, educational attainment, employment status, partisan attachment, having had COVID-19, number of doses administered of COVID-19 vaccine, and time spent reading the post. Significance levels are derived from linear regression models.

4 Differences of means in secondary specifications

Tables S9 to S14 expand on the results of the previous section with more detailed specifications. Tables S9 to S11 disaggregate confirmation framing effects by changing the vaccine brand mentioned in the fact-checking post. While, in general, results appear robust to the vaccine brand presented (Table S9), some differences emerge when they are broken down by partisan attachment (Tables S10 and S11). Consistent with the ***Hypothesis 3*** in the pre-approved plan, which described the independent pro- and counter-attitudinal effect of partisan attachment by type of vaccine, these results show a considerably higher relative effect for Moderna over Sputnik V and for AstraZeneca over Sputnik V among respondents who support the opposition, since in Argentina, the Sputnik V vaccine was typically associated with the government in the media. We expected that respondents who supported the government (i.e., *Frente de Todos (FdT)* voters) would be more inclined to share confirmations of Sputnik V than Moderna and AstraZeneca. We also expected that the opposition (i.e., voters of *Cambiemos*) would be more likely to share confirmation of Moderna than Sputnik and AtraZeneca. These effects have no associated cognitive cost. Differences in the overall effects on reactions between supporters of the government and the opposition are further detailed in section 5.1 of this Supplemental Information File.

Aside from the Argentina survey, the ones conducted in Brazil, Chile, and Colombia included different treatments by the use of printed 'TRUE' or 'FALSE' labels in the accompanying image. Tables S12 to S14 report differences in results by use of labels in each of the three surveys, extending the results from Table 2 in the main article. While the effects on emotions are stable to the inclusion or not of the label, engagement appears to be more volatile, increasing with the use of labels in the Brazil sample, decreasing in the Chile sample, and remaining about the same in the Colombia sample.

Table S9 Difference of means between the confirmation and refutation frames in the Argentina survey by brand of vaccine mentioned in the statement.

Variable	Overall	Moderna	Sputnik	AstraZeneca
Reactions				
Engage	0.188***	0.202***	0.177***	0.188***
	(0.018)	(0.032)	(0.032)	(0.031)
Like	0.163***	0.174***	0.157***	0.163***
	(0.015)	(0.027)	(0.026)	(0.025)
Share	0.042***	0.032	0.032	0.062**
	(0.012)	(0.020)	(0.023)	(0.021)
Comment	0.008	0.019	0.023	-0.020
	(0.009)	(0.014)	(0.015)	(0.016)
Emotions				
Optimistic	0.278***	0.250***	0.296***	0.296***
	(0.015)	(0.027)	(0.027)	(0.026)
Joyful	0.087***	0.099***	0.107***	0.057***
	(0.009)	(0.017)	(0.018)	(0.014)
Angry	-0.121***	-0.115***	-0.086***	-0.152***
	(0.012)	(0.021)	(0.021)	(0.022)
Sad	-0.035***	-0.047**	-0.030	-0.025
	(0.008)	(0.015)	(0.015)	(0.013)
Stressed	-0.072***	-0.070***	-0.083***	-0.066**
	(0.012)	(0.020)	(0.021)	(0.022)
Fearful	-0.015	-0.008	-0.018	-0.021
	(0.008)	(0.014)	(0.013)	(0.015)
Disgusted	-0.121***	-0.157***	-0.120***	-0.094***
	(0.014)	(0.023)	(0.025)	(0.024)
Indifferent	-0.028	-0.009	-0.052	-0.029
	(0.020)	(0.035)	(0.036)	(0.036)

Note: Robust standard errors in parentheses. *** $p < 0.001$, ** $p < 0.01$, * $p < 0.05$. Each cell corresponds to a different regression using as sample the branch of the treatment indicated in the header. Coefficients represent the effect of the confirmation frame on the reaction or emotion indicated in the first column compared against the refutation frame. All regressions control for age, sex, educational attainment, employment status, partisan attachment, having had COVID-19, number of doses administered of COVID-19 vaccine, and time spent reading the post.

Table S10 Difference of means in reactions between the confirmation and refutation frames in the Argentina survey by brand of vaccine mentioned in the statement and partisan attachment of the respondent.

Variable	Overall	Moderna	Sputnik	AstraZeneca
Government (Frente de Todos)				
Engage	0.269***	0.363***	0.310***	0.200**
	(0.037)	(0.067)	(0.066)	(0.066)
Like	0.238***	0.315***	0.222***	0.241***
	(0.034)	(0.064)	(0.062)	(0.060)
Share	0.057*	0.069	0.094	0.005
	(0.027)	(0.042)	(0.052)	(0.045)
Comment	0.024	0.011	0.072*	-0.014
	(0.017)	(0.027)	(0.031)	(0.033)
Opposition (Juntos por el Cambio)				
Engage	0.160***	0.205***	0.072	0.198***
	(0.027)	(0.046)	(0.050)	(0.048)
Like	0.135***	0.166***	0.103**	0.146***
	(0.021)	(0.039)	(0.037)	(0.037)
Share	0.042*	0.048	-0.017	0.079*
	(0.018)	(0.027)	(0.035)	(0.031)
Comment	0.004	0.016	0.007	-0.005
	(0.014)	(0.022)	(0.023)	(0.027)
None (blank vote)				
Engage	0.163***	0.097	0.240***	0.160**
	(0.032)	(0.059)	(0.059)	(0.054)
Like	0.141***	0.100*	0.200***	0.131**
	(0.026)	(0.049)	(0.050)	(0.042)
Share	0.032	-0.023	0.064	0.068*
	(0.021)	(0.041)	(0.038)	(0.034)
Comment	0.001	0.021	0.016	-0.040
	(0.013)	(0.019)	(0.021)	(0.024)

Note: Robust standard errors in parentheses. *** $p < 0.001$, ** $p < 0.01$, * $p < 0.05$. Each cell corresponds to a different regression using as sample the branch of the treatment indicated in the header. Coefficients represent the effect of the confirmation frame on the reaction indicated in the first column compared against the refutation frame. All regressions control for age, sex, educational attainment, employment status, partisan attachment, having had COVID-19, number of doses administered of COVID-19 vaccine, and time spent reading the post.

Table S11 Difference of means in emotions generation between the confirmation and refutation frames in the Argentina survey by brand of vaccine mentioned in the statement and partisan attachment of the respondent.

Variable	Overall	Moderna	Sputnik	AstraZeneca
Government (Frente de Todos)				
Optimistic	0.377***	0.361***	0.404***	0.404***
	(0.034)	(0.064)	(0.063)	(0.059)
Joyful	0.118***	0.128**	0.092*	0.125**
	(0.023)	(0.045)	(0.042)	(0.040)
Angry	-0.177***	-0.204***	-0.150**	-0.190***
	(0.027)	(0.054)	(0.046)	(0.046)
Sad	-0.052**	-0.091*	-0.044	-0.027
	(0.017)	(0.040)	(0.027)	(0.016)
Stressed	-0.102***	-0.108**	-0.100**	-0.123**
	(0.023)	(0.041)	(0.037)	(0.045)
Fearful	-0.018	-0.011	-0.019	-0.030
	(0.011)	(0.023)	(0.014)	(0.023)
Disgusted	-0.159***	-0.151***	-0.174***	-0.158***
	(0.025)	(0.043)	(0.045)	(0.046)
Indifferent	0.005	0.004	0.010	0.001
	(0.039)	(0.068)	(0.068)	(0.066)
Opposition (Juntos por el Cambio)				
Optimistic	0.266***	0.296***	0.266***	0.249***
	(0.021)	(0.037)	(0.039)	(0.038)
Joyful	0.077***	0.076***	0.100***	0.062**
	(0.013)	(0.022)	(0.025)	(0.022)
Angry	-0.115***	-0.102**	-0.074*	-0.168***
	(0.019)	(0.033)	(0.034)	(0.035)
Sad	-0.026*	-0.047*	-0.003	-0.016
	(0.012)	(0.021)	(0.022)	(0.019)
Stressed	-0.094***	-0.087**	-0.111***	-0.080*
	(0.018)	(0.028)	(0.031)	(0.034)
Fearful	-0.011	0.004	-0.017	-0.028
	(0.012)	(0.019)	(0.022)	(0.023)
Disgusted	-0.118***	-0.185***	-0.092*	-0.092*
	(0.022)	(0.036)	(0.039)	(0.039)
Indifferent	-0.014	0.009	-0.044	-0.024
	(0.031)	(0.053)	(0.056)	(0.056)

None (blank vote)				
Optimistic	0.213***	0.097*	0.282***	0.256***
	(0.027)	(0.049)	(0.045)	(0.045)
Joyful	0.066***	0.112***	0.089**	-0.013
	(0.015)	(0.031)	(0.033)	(0.014)
Angry	-0.086***	-0.075*	-0.042	-0.106**
	(0.020)	(0.033)	(0.033)	(0.040)
Sad	-0.033*	-0.023	-0.038	-0.020
	(0.016)	(0.026)	(0.029)	(0.028)
Stressed	-0.017	-0.006	-0.038	-0.008
	(0.022)	(0.035)	(0.045)	(0.039)
Fearful	-0.017	-0.028	-0.005	-0.004
	(0.017)	(0.031)	(0.031)	(0.030)
Disgusted	-0.095***	-0.118**	-0.128*	-0.070
	(0.026)	(0.044)	(0.053)	(0.046)
Indifferent	-0.061	-0.033	-0.115	-0.026
	(0.038)	(0.069)	(0.070)	(0.067)

Note: Robust standard errors in parentheses. *** $p < 0.001$, ** $p < 0.01$, * $p < 0.05$. Each cell corresponds to a different regression using as sample the branch of the treatment indicated in the header. Coefficients represent the effect of the confirmation frame on the emotion indicated in the first column compared against the refutation frame. All regressions control for age, sex, educational attainment, employment status, partisan attachment, having had COVID-19, number of doses administered of COVID-19 vaccine, and time spent reading the post.

Table S12 Difference of means between the confirmation and refutation frames in the Brazil survey by inclusion or not of 'TRUE'/'FALSE' label in the picture.

Variable	Overall	With label	Without label
Reactions			
Engage	0.110***	0.163***	0.089**
	(0.020)	(0.034)	(0.034)
Like	0.108***	0.164***	0.088**
	(0.018)	(0.032)	(0.032)
Share	0.000	-0.014	0.002
	(0.014)	(0.025)	(0.025)
Comment	-0.003	0.009	-0.015
	(0.013)	(0.020)	(0.022)
Emotions			
Optimistic	0.172***	0.274***	0.230***
	(0.017)	(0.030)	(0.031)
Joyful	0.066***	0.127***	0.087***
	(0.014)	(0.025)	(0.025)
Angry	-0.062***	-0.079***	-0.081***
	(0.011)	(0.020)	(0.018)
Sad	-0.078***	-0.133***	-0.078***
	(0.012)	(0.021)	(0.018)
Stressed	-0.034***	-0.061***	-0.036*
	(0.009)	(0.017)	(0.017)
Fearful	-0.042***	-0.060**	-0.039*
	(0.010)	(0.020)	(0.019)
Disgusted	-0.073***	-0.119***	-0.107***
	(0.012)	(0.021)	(0.020)
Indifferent	-0.010	-0.031	-0.026
	(0.020)	(0.033)	(0.032)

Note: Robust standard errors in parentheses. *** $p < 0.001$, ** $p < 0.01$, * $p < 0.05$. Each cell corresponds to a different regression using as sample the branch of the treatment indicated in the header. Coefficients represent the effect of the confirmation frame on the reaction or emotion indicated in the first column compared against the refutation frame. All regressions control for age, sex, educational attainment, employment status, partisan attachment, having had COVID-19, number of doses administered of COVID-19 vaccine, and time spent reading the post.

Table S13 Difference of means between the confirmation and refutation frames in the Chile survey by inclusion or not of 'TRUE'/'FALSE' label in the picture.

Variable	Overall	With label	Without label
Reactions			
Engage	0.133***	0.104**	0.199***
	(0.019)	(0.032)	(0.032)
Like	0.140***	0.158***	0.185***
	(0.016)	(0.025)	(0.027)
Share	0.014	-0.035	0.056*
	(0.014)	(0.024)	(0.024)
Comment	0.007	0.003	0.004
	(0.011)	(0.015)	(0.014)
Emotions			
Optimistic	0.178***	0.228***	0.270***
	(0.015)	(0.027)	(0.029)
Joyful	0.075***	0.078***	0.132***
	(0.011)	(0.016)	(0.021)
Angry	-0.063***	-0.102***	-0.063**
	(0.012)	(0.023)	(0.020)
Sad	-0.008	-0.021	-0.028*
	(0.010)	(0.012)	(0.013)
Stressed	-0.022*	-0.027	-0.022
	(0.011)	(0.023)	(0.020)
Fearful	-0.032**	-0.038	-0.046*
	(0.011)	(0.021)	(0.021)
Disgusted	-0.034**	-0.039	-0.050*
	(0.011)	(0.024)	(0.020)
Indifferent	-0.075***	-0.065	-0.140***
	(0.020)	(0.036)	(0.034)

Note: Robust standard errors in parentheses. *** $p < 0.001$, ** $p < 0.01$, * $p < 0.05$. Each cell corresponds to a different regression using as sample the branch of the treatment indicated in the header. Coefficients represent the effect of the confirmation frame on the reaction or emotion indicated in the first column compared against the refutation frame. All regressions control for age, sex, educational attainment, employment status, partisan attachment, having had COVID-19, number of doses administered of COVID-19 vaccine, and time spent reading the post.

Table S14 Difference of means between the confirmation and refutation frames in the Colombia survey by inclusion or not of 'TRUE'/'FALSE' label in the picture.

Variable	Overall	With label	Without label
Reactions			
Engage	0.129***	0.153***	0.138***
	(0.020)	(0.034)	(0.034)
Like	0.088***	0.111***	0.127***
	(0.017)	(0.027)	(0.029)
Share	0.046**	0.037	0.035
	(0.015)	(0.027)	(0.027)
Comment	0.029*	0.034	0.015
	(0.013)	(0.018)	(0.018)
Emotions			
Optimistic	0.159***	0.243***	0.198***
	(0.017)	(0.030)	(0.031)
Joyful	0.054***	0.057**	0.082***
	(0.011)	(0.018)	(0.019)
Angry	-0.015	-0.063**	-0.035*
	(0.011)	(0.019)	(0.017)
Sad	-0.030**	-0.053**	-0.042**
	(0.012)	(0.017)	(0.015)
Stressed	-0.020	-0.034	-0.017
	(0.010)	(0.022)	(0.020)
Fearful	-0.021	-0.009	-0.063*
	(0.013)	(0.027)	(0.025)
Disgusted	-0.003	-0.015	0.019
	(0.008)	(0.017)	(0.014)
Indifferent	-0.103***	-0.135***	-0.125***
	(0.020)	(0.036)	(0.035)

Note: Robust standard errors in parentheses. *** $p < 0.001$, ** $p < 0.01$, * $p < 0.05$. Each cell corresponds to a different regression using as sample the branch of the treatment indicated in the header. Coefficients represent the effect of the confirmation frame on the reaction or emotion indicated in the first column compared against the refutation frame. All regressions control for age, sex, educational attainment, employment status, partisan attachment, having had COVID-19, number of doses administered of COVID-19 vaccine, and time spent reading the post.

5 Heterogeneity in results

Tables S15 to S27 present several disaggregations of the confirmation framing effect across political preferences, education, and attention, with the aim of exploring hypotheses about how this effect may or not depend on the issue salience, prior beliefs, or cognitive mechanisms.

Tables S15 and S16 summarize some confirmation framing effects on reactions and emotions generation across partisan attachment, educational attainment and time spent reading the question, by survey. More detailed information on the heterogeneity of effects in these dimensions is presented in Tables S17 to S28. In addition to the target variable for potential heterogeneity, all estimates are obtained controlling for socio-demographic variables (gender, age, educational attainment, and employment status), partisan attachment, having had COVID-19, number of doses administered of COVID-19 vaccine, and time spent reading the question.

Table S15 Summary of heterogeneous effects on reactions in the general specification by country and selected dimensions. Difference of means of emotions generation by confirmation and refutation frame assignment.

	Partisan attachment		Completed	Incomplete	Time to read	
Variable	Government	Opposition	secondary (or -)	college (or +)	First quintile	Fifth quintile
Argentina						
Engage	0.267***	0.160***	0.240***	0.171***	0.158***	0.248***
Like	0.238***	0.135***	0.169***	0.161***	0.132***	0.221***
Share	0.054*	0.043*	0.070**	0.031*	0.036	0.076*
Comment	0.025	0.004	0.031	0.001	-0.006	-0.004
Brazil						
Engage	0.109**	0.171***	0.125**	0.137***	0.088	0.149**
Like	0.095**	0.179***	0.131***	0.128***	0.074	0.087
Share	0.015	-0.001	-0.011	0.008	0.011	0.009
Comment	0.020	-0.027	-0.025	0.015	-0.022	0.047
Chile						
Engage	0.153***	0.163***	0.158**	0.149***	0.229***	0.159**
Like	0.171***	0.191***	0.140***	0.179***	0.212***	0.112**
Share	-0.009	0.029	0.004	0.012	0.012	0.018
Comment	0.006	0.005	0.010	0.004	0.007	0.066*
Colombia						
Engage	0.150***	0.158**	0.138**	0.147***	0.208**	0.118*
Like	0.116***	0.197***	0.160***	0.108***	0.189***	0.082*
Share	0.040	0.011	-0.015	0.049*	0.048	0.073
Comment	0.033	-0.014	0.031	0.022	-0.015	0.020

Note: Robust standard errors in parentheses. *** $p < 0.001$, ** $p < 0.01$, * $p < 0.05$. Each cell corresponds to a different regression using only the restricted sample indicated in the header. Coefficients represent the effect of the confirmation frame on the reaction indicated in the first column compared against the refutation frame. All regressions control for age, sex, educational attainment, employment status, partisan attachment, having had COVID-19, number of doses administered of COVID-19 vaccine, and time spent reading the post.

Table S16 Summary of heterogeneous effects on emotions generation in the general specification by country and selected dimensions. Difference of means of emotions generation by confirmation and refutation frame assignment.

	Partisan attachment		Completed	Incomplete	Time to read	
Variable	Government	Opposition	secondary (or -)	college (or +)	First quintile	Fifth quintile
Argentina						
Optimistic	0.377***	0.266***	0.252***	0.288***	0.206***	0.372***
Joyful	0.118***	0.077***	0.084***	0.088***	0.082***	0.062**
Angry	-0.177***	-0.115***	-0.091***	-0.131***	-0.112***	-0.121***
Sad	-0.052**	-0.026*	-0.041*	-0.033***	-0.022	-0.030
Stressed	-0.102***	-0.094***	-0.052	-0.080***	-0.056*	-0.090***
Fearful	-0.018	-0.011	-0.015	-0.014	-0.019	-0.017
Disgusted	-0.159***	-0.118***	-0.084**	-0.136***	-0.124***	-0.155***
Indifferent	0.005	-0.014	-0.042	-0.022	0.016	-0.061
Brazil						
Optimistic	0.174***	0.343***	0.165***	0.316***	0.194***	0.230***
Joyful	0.071**	0.161***	0.066*	0.132***	0.069	0.133***
Angry	-0.043	-0.127***	-0.071***	-0.093***	-0.082**	-0.079**
Sad	-0.058**	-0.157***	-0.101***	-0.106***	-0.096**	-0.108**
Stressed	-0.033	-0.093***	-0.027	-0.065***	-0.094**	-0.016
Fearful	-0.029	-0.072***	-0.027	-0.066***	-0.055	-0.056
Disgusted	-0.034	-0.185***	-0.081***	-0.132***	-0.135***	-0.136***
Indifferent	-0.067	0.005	-0.005	-0.042	0.037	-0.006
Chile						
Optimistic	0.245***	0.303***	0.159***	0.270***	0.257***	0.247***
Joyful	0.100***	0.139***	0.068**	0.115***	0.137***	0.150***
Angry	-0.096***	-0.069**	-0.083*	-0.083***	-0.066*	-0.106**
Sad	-0.013	-0.026	-0.023	-0.024*	-0.019	-0.028
Stressed	-0.037	-0.033	-0.016	-0.027	-0.085*	0.014
Fearful	-0.012	-0.075**	-0.073	-0.036*	-0.059*	-0.025
Disgusted	-0.069**	-0.028	0.021	-0.068***	-0.051	-0.030

Indifferent	-0.092*	-0.129**	-0.010	-0.120***	-0.115	-0.134*
Colombia						
Optimistic	0.245***	0.209***	0.237***	0.220***	0.210***	0.178***
Joyful	0.088***	0.085*	0.070*	0.069***	0.052	0.067*
Angry	-0.063***	-0.042	-0.032	-0.056***	-0.045	-0.045
Sad	-0.061***	-0.057*	-0.037	-0.053***	0.000	-0.048
Stressed	-0.048*	0.016	0.021	-0.049**	0.042	-0.029
Fearful	-0.012	-0.084	-0.027	-0.040	-0.026	-0.026
Disgusted	-0.018	0.008	0.043	-0.019	0.002	0.040
Indifferent	-0.141***	-0.064	-0.207***	-0.101***	-0.186**	-0.151**

Note: Robust standard errors in parentheses. *** $p < 0.001$, ** $p < 0.01$, * $p < 0.05$. Each cell corresponds to a different regression using only the restricted sample indicated in the header. Coefficients represent the effect of the confirmation frame on the emotion indicated in the first column compared against the refutation frame. All regressions control for age, sex, educational attainment, employment status, partisan attachment, having had COVID-19, number of doses administered of COVID-19 vaccine, and time spent reading the post.

5.1 Partisan attachment and vaccination status

Tables S17 through S22 provide estimates of heterogeneous effects by political preferences and vaccination status. These results show a slight pattern of more pronounced effects for those who were expected to be more supportive of vaccination. For instance, a higher preference was expected from voters of the incumbent administration of Alberto Fernandez in Argentina, who actively supported quarantine measures early in the pandemic and made vaccination a policy priority. While partisan polarization is high in Argentina, most political actors supported widespread vaccination and partisan conflict centered on the quality of the government's response to the pandemic. Both the leading opposition coalition voters (*Cambiemos*) and independent voters (blank vote) shared the confirmation frames at higher rates than the refutation frames, but the magnitude of the effect was significantly surpassed in the sample of government supporters (Tables S17 and S19). In the Argentine election survey, 1,080 respondents indicated a preference for *Cambiemos*, 613 voters indicated a preference for the *Frente de Todos*, and 727 respondents indicated a preference for blank voting (see Figure S5). These results are complementary to those presented in section 4 on differential effects by vaccine brand and partisan attachment in the Argentina survey. In line with this case, the Brazil survey was conducted during the presidency of Jair Bolsonrao, who openly opposed COVID-19 vaccination. As expected, his supporters show less sensitivity to the framing effect on engagement, although this difference is not statistically significant (Table S17).

The results presented in Tables S21 and S22 also show that the confirmation frame induces higher engagement and a higher rate of likes among respondents with higher vaccine doses. A caveat here is that people with two or more doses represent about 85% to 95% of the survey sample for each country, with Colombia being the one with the fewest respondents vaccinated twice or more times. This is consistent with a valence interpretation of the frame effect and inconsistent with the cognitive burden hypothesis, as discussed in the main article. Results show that the effect of the confirmation frame is more strongly associated with more positive emotions and less negative ones among respondents with 2 or more COVID-19 doses.

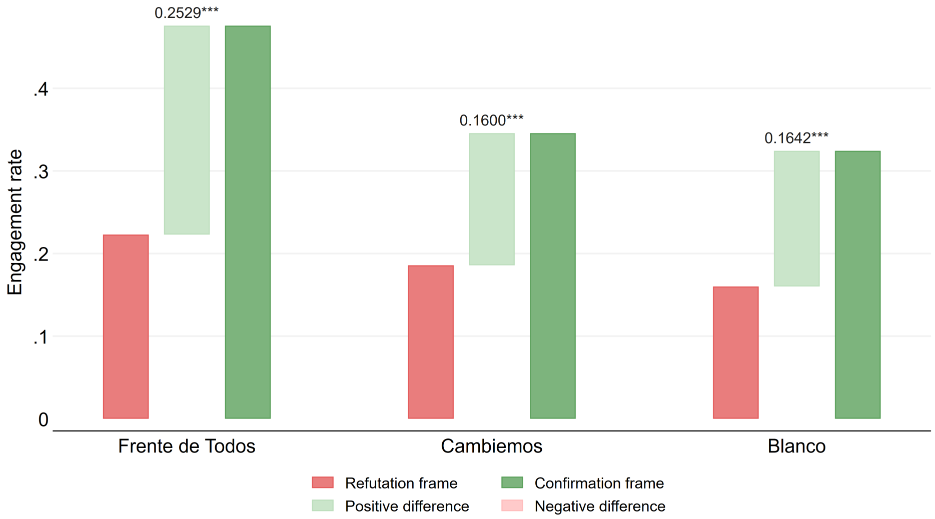

Figure S5 Engagement rates for the confirmation ('TRUE') and refutation ('FALSE') frames by vote preference. Exposure to the confirmation frame has a larger effect among supporters of the incumbent President Alberto Fernandez (*Frente de Todos, FdT*). Supporters of the opposition (*Cambiemos*) and the independent voters (*Blanco*) also display a larger intent to like, share, or comment the confirmation frame, but the effect is more modest.

Table S17 Heterogeneity in effects on reactions in the general specification by partisan attachment and country. Difference of means of reactions by confirmation and refutation frame assignment, partisan attachment, and country.

Variable	Government	Opposition	Blank vote	Oppos. v. gov.
Argentina				
Engage	0.269***	0.160***	0.163***	-0.102*
	(0.037)	(0.027)	(0.032)	(0.046)
Like	0.238***	0.135***	0.141***	-0.097*
	(0.034)	(0.021)	(0.026)	(0.039)
Share	0.057*	0.042*	0.032	-0.010
	(0.027)	(0.018)	(0.021)	(0.032)
Comment	0.024	0.004	0.001	-0.023
	(0.017)	(0.014)	(0.013)	(0.022)
Brazil				
Engage	0.107**	0.170***	0.099	0.061
	(0.038)	(0.037)	(0.056)	(0.053)
Like	0.095**	0.179***	0.098	0.079
	(0.032)	(0.037)	(0.050)	(0.049)
Share	0.011	0.000	-0.024	-0.014
	(0.024)	(0.032)	(0.037)	(0.039)
Comment	0.023	-0.031	0.002	-0.048

	(0.026)	(0.022)	(0.032)	(0.034)
Chile				
Engage	0.150***	0.163***	0.147**	0.032
	(0.034)	(0.040)	(0.049)	(0.052)
Like	0.169***	0.190***	0.155***	0.034
	(0.030)	(0.030)	(0.038)	(0.042)
Share	-0.009	0.028	0.021	0.039
	(0.023)	(0.031)	(0.040)	(0.039)
Comment	0.004	0.004	0.005	0.001
	(0.013)	(0.020)	(0.021)	(0.024)
Colombia				
Engage	0.151***	0.155**	0.124**	0.010
	(0.035)	(0.058)	(0.043)	(0.067)
Like	0.115***	0.197***	0.064	0.078
	(0.029)	(0.045)	(0.034)	(0.053)
Share	0.040	0.008	0.038	-0.026
	(0.026)	(0.051)	(0.033)	(0.056)
Comment	0.032	-0.014	0.030	-0.038
	(0.018)	(0.031)	(0.021)	(0.036)

Note: Robust standard errors in parentheses. *** p < 0.001, ** p < 0.01, * p < 0.05. Each cell in the first three columns corresponds to a different regression using only the restricted sample indicated in the header. Coefficients represent the effect of the confirmation frame on the reaction indicated in the first column compared against the refutation frame. The last column present the difference between coefficients in the second and first columns from the interaction of the treatment with the categories indicated. All regressions control for age, sex, educational attainment, employment status, partisan attachment, having had COVID-19, number of doses administered of COVID-19 vaccine, and time spent reading the post.

Table S18 Heterogeneity in effects on emotions generation in the general specification by partisan attachment and country. Difference of means of emotions generation by confirmation and refutation frame assignment, partisan attachment, and country.

Variable	Government	Opposition	Blank vote	Oppos. v. gov.
Argentina				
Optimistic	0.377***	0.266***	0.213***	-0.106**
	(0.034)	(0.021)	(0.027)	(0.040)
Joyful	0.118***	0.077***	0.066***	-0.045
	(0.023)	(0.013)	(0.015)	(0.027)
Angry	-0.177***	-0.115***	-0.086***	0.059
	(0.027)	(0.019)	(0.020)	(0.033)
Sad	-0.052**	-0.026*	-0.033*	0.024

	(0.017)	(0.012)	(0.016)	(0.020)
Stressed	-0.102***	-0.094***	-0.017	0.010
	(0.023)	(0.018)	(0.022)	(0.029)
Fearful	-0.018	-0.011	-0.017	0.010
	(0.011)	(0.012)	(0.017)	(0.017)
Disgusted	-0.159***	-0.118***	-0.095***	0.044
	(0.025)	(0.022)	(0.026)	(0.033)
Indifferent	0.005	-0.014	-0.061	-0.018
	(0.039)	(0.031)	(0.038)	(0.049)
Brazil				
Optimistic	0.174***	0.343***	0.252***	0.166***
	(0.032)	(0.035)	(0.051)	(0.047)
Joyful	0.071**	0.161***	0.069	0.090*
	(0.024)	(0.031)	(0.040)	(0.039)
Angry	-0.043	-0.127***	-0.085**	-0.078*
	(0.024)	(0.021)	(0.030)	(0.031)
Sad	-0.058**	-0.157***	-0.085**	-0.100**
	(0.022)	(0.022)	(0.031)	(0.031)
Stressed	-0.033	-0.093***	0.003	-0.061*
	(0.020)	(0.019)	(0.022)	(0.028)
Fearful	-0.029	-0.072***	-0.054	-0.036
	(0.024)	(0.020)	(0.030)	(0.031)
Disgusted	-0.034	-0.185***	-0.107***	-0.149***
	(0.021)	(0.024)	(0.030)	(0.032)
Indifferent	-0.067	0.005	-0.041	0.070
	(0.040)	(0.031)	(0.057)	(0.050)
Chile				
Optimistic	0.245***	0.303***	0.176***	0.061
	(0.031)	(0.034)	(0.041)	(0.046)
Joyful	0.100***	0.139***	0.075**	0.032
	(0.021)	(0.025)	(0.025)	(0.032)
Angry	-0.096***	-0.069**	-0.069*	0.032
	(0.022)	(0.025)	(0.032)	(0.034)
Sad	-0.013	-0.026	-0.046*	-0.013
	(0.012)	(0.014)	(0.021)	(0.018)
Stressed	-0.037	-0.033	0.010	0.004
	(0.023)	(0.026)	(0.032)	(0.035)
Fearful	-0.012	-0.075**	-0.057	-0.066*
	(0.022)	(0.025)	(0.034)	(0.032)
Disgusted	-0.069**	-0.028	-0.034	0.045

	(0.023)	(0.026)	(0.034)	(0.035)
Indifferent	-0.092*	-0.129**	-0.085	-0.039
	(0.038)	(0.042)	(0.055)	(0.056)
Colombia				
Optimistic	0.245***	0.209***	0.176***	-0.015
	(0.031)	(0.054)	(0.038)	(0.062)
Joyful	0.088***	0.085*	0.032	0.006
	(0.019)	(0.033)	(0.020)	(0.038)
Angry	-0.063***	-0.042	-0.036	0.022
	(0.018)	(0.030)	(0.025)	(0.036)
Sad	-0.061***	-0.057*	-0.019	0.007
	(0.015)	(0.024)	(0.024)	(0.029)
Stressed	-0.048*	0.016	-0.018	0.059
	(0.021)	(0.032)	(0.031)	(0.039)
Fearful	-0.012	-0.084	-0.068	-0.061
	(0.025)	(0.044)	(0.036)	(0.050)
Disgusted	-0.018	0.008	0.032	0.035
	(0.014)	(0.025)	(0.023)	(0.031)
Indifferent	-0.141***	-0.064	-0.135**	0.052
	(0.036)	(0.058)	(0.046)	(0.067)

Note: Robust standard errors in parentheses. *** p < 0.001, ** p < 0.01, * p < 0.05. Each cell in the first three columns corresponds to a different regression using only the restricted sample indicated in the header. Coefficients represent the effect of the confirmation frame on the emotion indicated in the first column compared against the refutation frame. The last column present the difference between coefficients in the second and first columns from the interaction of the treatment with the categories indicated. All regressions control for age, sex, educational attainment, employment status, partisan attachment, having had COVID-19, number of doses administered of COVID-19 vaccine, and time spent reading the post.

Table S19 Heterogeneity in levels of reactions in the general specification by partisan attachment and country. Levels of reactions by confirmation and refutation frame assignment, partisan attachment, and country.

	Government		Opposition	
Variable	Refutation	Confirmation	Refutation	Confirmation
Argentina				
Engage	0.223	0.476	0.186	0.346
	(0.024)	(0.029)	(0.017)	(0.021)
Like	0.108	0.345	0.073	0.210
	(0.018)	(0.028)	(0.012)	(0.018)
Share	0.102	0.145	0.069	0.113

	(0.017)	(0.021)	(0.011)	(0.014)
Comment	0.045	0.066	0.051	0.052
	(0.012)	(0.015)	(0.010)	(0.010)
Brazil				
Engage	0.306	0.406	0.533	0.693
	(0.026)	(0.028)	(0.027)	(0.025)
Like	0.164	0.258	0.398	0.561
	(0.021)	(0.025)	(0.026)	(0.026)
Share	0.085	0.101	0.219	0.214
	(0.016)	(0.017)	(0.022)	(0.022)
Comment	0.110	0.126	0.107	0.087
	(0.018)	(0.019)	(0.017)	(0.015)
Chile				
Engage	0.218	0.368	0.234	0.407
	(0.022)	(0.026)	(0.025)	(0.030)
Like	0.117	0.284	0.059	0.255
	(0.017)	(0.024)	(0.014)	(0.027)
Share	0.112	0.107	0.134	0.167
	(0.017)	(0.016)	(0.020)	(0.023)
Comment	0.032	0.039	0.052	0.057
	(0.009)	(0.010)	(0.013)	(0.014)
Colombia				
Engage	0.284	0.427	0.346	0.507
	(0.022)	(0.026)	(0.037)	(0.043)
Like	0.141	0.254	0.086	0.287
	(0.017)	(0.023)	(0.022)	(0.039)
Share	0.131	0.172	0.228	0.235
	(0.017)	(0.020)	(0.033)	(0.037)
Comment	0.044	0.076	0.080	0.081
	(0.010)	(0.014)	(0.021)	(0.023)

Note: Standard errors in parentheses. Each column presents the average value of the reaction indicated in the first column for the restricted sample and the refutation or confirmation frame indicated in the header.

Table S20 Heterogeneity in levels of emotions generation in the general specification by partisan attachment and country. Levels of emotions generation by confirmation and refutation frame assignment, partisan attachment, and country.

	Government		Opposition	
Variable	Refutation	Confirmation	Refutation	Confirmation

Argentina				
Optimistic	0.099	0.469	0.038	0.303
	(0.017)	(0.029)	(0.008)	(0.020)
Joyful	0.035	0.155	0.010	0.087
	(0.010)	(0.021)	(0.004)	(0.012)
Angry	0.220	0.048	0.158	0.048
	(0.023)	(0.013)	(0.016)	(0.009)
Sad	0.073	0.021	0.045	0.022
	(0.015)	(0.008)	(0.009)	(0.006)
Stressed	0.137	0.038	0.140	0.048
	(0.019)	(0.011)	(0.015)	(0.009)
Fearful	0.035	0.010	0.043	0.032
	(0.010)	(0.006)	(0.009)	(0.008)
Disgusted	0.191	0.034	0.206	0.087
	(0.022)	(0.011)	(0.018)	(0.012)
Indifferent	0.331	0.328	0.462	0.439
	(0.027)	(0.028)	(0.022)	(0.021)
Brazil				
Optimistic	0.117	0.302	0.242	0.583
	(0.018)	(0.026)	(0.023)	(0.026)
Joyful	0.063	0.138	0.135	0.290
	(0.014)	(0.019)	(0.018)	(0.024)
Angry	0.123	0.072	0.138	0.020
	(0.018)	(0.015)	(0.019)	(0.007)
Sad	0.107	0.047	0.170	0.017
	(0.017)	(0.012)	(0.020)	(0.007)
Stressed	0.088	0.050	0.118	0.023
	(0.016)	(0.012)	(0.017)	(0.008)
Fearful	0.123	0.082	0.104	0.039
	(0.018)	(0.015)	(0.016)	(0.010)
Disgusted	0.095	0.057	0.205	0.025
	(0.016)	(0.013)	(0.022)	(0.008)
Indifferent	0.451	0.399	0.219	0.223
	(0.028)	(0.028)	(0.022)	(0.022)
Chile				
Optimistic	0.117	0.357	0.079	0.380
	(0.017)	(0.025)	(0.016)	(0.030)
Joyful	0.034	0.138	0.028	0.160
	(0.010)	(0.018)	(0.010)	(0.023)

Angry	0.155	0.053	0.145	0.080
	(0.019)	(0.012)	(0.021)	(0.017)
Sad	0.034	0.017	0.045	0.019
	(0.010)	(0.007)	(0.012)	(0.008)
Stressed	0.117	0.079	0.124	0.087
	(0.017)	(0.014)	(0.019)	(0.017)
Fearful	0.086	0.081	0.138	0.061
	(0.015)	(0.015)	(0.020)	(0.015)
Disgusted	0.149	0.076	0.117	0.103
	(0.019)	(0.014)	(0.019)	(0.019)
Indifferent	0.473	0.385	0.517	0.388
	(0.027)	(0.026)	(0.029)	(0.030)
Colombia				
Optimistic	0.124	0.362	0.198	0.426
	(0.016)	(0.026)	(0.031)	(0.043)
Joyful	0.029	0.113	0.037	0.132
	(0.008)	(0.017)	(0.015)	(0.029)
Angry	0.100	0.040	0.099	0.059
	(0.015)	(0.010)	(0.024)	(0.020)
Sad	0.075	0.017	0.080	0.029
	(0.013)	(0.007)	(0.021)	(0.015)
Stressed	0.109	0.065	0.080	0.096
	(0.015)	(0.013)	(0.021)	(0.025)
Fearful	0.146	0.138	0.210	0.140
	(0.017)	(0.018)	(0.032)	(0.030)
Disgusted	0.053	0.034	0.049	0.066
	(0.011)	(0.010)	(0.017)	(0.021)
Indifferent	0.551	0.407	0.420	0.331
	(0.025)	(0.026)	(0.039)	(0.040)

Note: Standard errors in parentheses. Each column presents the average value of the reaction indicated in the first column for the restricted sample and the refutation or confirmation frame indicated in the header.

Table S21 Heterogeneity in effects on reactions in the general specification by vaccination status and country. Difference of means of reactions by confirmation and refutation frame assignment, vaccination status, and country.

Variable	Non-vaccinated†	Vaccinated once†	Vaccinated twice (or +)
Argentina			
Engage	-0.069	-0.025	0.210***

	(0.073)	(0.094)	(0.019)
Like	-0.034	0.034	0.179***
	(0.047)	(0.076)	(0.016)
Share	-0.036	-0.032	0.051***
	(0.037)	(0.067)	(0.013)
Comment	-0.003	-0.000	0.007
	(0.048)	(0.000)	(0.009)
Brazil			
Engage	-0.054	0.044	0.151***
	(0.115)	(0.151)	(0.025)
Like	-0.024	0.071	0.141***
	(0.106)	(0.130)	(0.023)
Share	-0.130	0.046	0.004
	(0.080)	(0.119)	(0.019)
Comment	-0.001	-0.210	0.007
	(0.045)	(0.106)	(0.016)
Chile			
Engage	0.010	0.033	0.154***
	(0.125)	(0.178)	(0.024)
Like	-0.015	-0.015	0.180***
	(0.023)	(0.198)	(0.019)
Share	-0.031	0.088	0.006
	(0.076)	(0.104)	(0.017)
Comment	0.055	0.138	0.001
	(0.095)	(0.270)	(0.010)
Colombia			
Engage	-0.038	0.103	0.153***
	(0.100)	(0.077)	(0.027)
Like	0.053	0.088	0.122***
	(0.035)	(0.058)	(0.022)
Share	-0.064	0.033	0.039
	(0.067)	(0.053)	(0.022)
Comment	-0.053	0.044	0.026*
	(0.078)	(0.056)	(0.012)

Note: Robust standard errors in parentheses. *** $p < 0.001$, ** $p < 0.01$, * $p < 0.05$. Each cell corresponds to a different regression using only the restricted sample indicated in the header. Coefficients represent the effect of the confirmation frame on the reaction indicated in the first column compared against the refutation frame. All regressions control for age, sex, educational attainment, employment status, partisan attachment, having had COVID-19, number of doses administered of COVID-19 vaccine, and time spent reading the post.

† This category comprises around or less than 10% of the observations.

Table S22 Heterogeneity in effects on emotions in the general specification by vaccination status and country. Difference of means of reactions by confirmation and refutation frame assignment, vaccination status, and country.

Variable	Non-vaccinated†	Vaccinated once†	Vaccinated twice (or +)
Argentina			
Optimistic	0.007	0.089	0.300***
	(0.032)	(0.076)	(0.016)
Joyful	0.047	-0.000	0.092***
	(0.029)	(0.000)	(0.010)
Angry	-0.086	0.003	-0.131***
	(0.063)	(0.077)	(0.013)
Sad	0.032	0.000	-0.042***
	(0.025)		(0.009)
Stressed	-0.017	0.042	-0.077***
	(0.059)	(0.091)	(0.012)
Fearful	0.025	-0.010	-0.018*
	(0.044)	(0.056)	(0.008)
Disgusted	-0.205*	0.004	-0.123***
	(0.084)	(0.092)	(0.014)
Indifferent	0.136	-0.041	-0.034
	(0.082)	(0.116)	(0.021)
Brazil			
Optimistic	0.181*	0.120	0.270***
	(0.075)	(0.087)	(0.023)
Joyful	-0.006	0.047	0.116***
	(0.058)	(0.056)	(0.019)
Angry	-0.142	0.026	-0.091***
	(0.085)	(0.115)	(0.014)
Sad	-0.024	-0.096	-0.109***
	(0.049)	(0.111)	(0.014)
Stressed	-0.051	0.057	-0.056***
	(0.076)	(0.121)	(0.012)
Fearful	-0.229**	-0.035	-0.045***
	(0.077)	(0.142)	(0.014)
Disgusted	-0.012	-0.030	-0.119***
	(0.095)	(0.120)	(0.015)
Indifferent	0.131	-0.113	-0.033
	(0.118)	(0.130)	(0.024)
Chile			

Optimistic	0.046	0.177	0.256***
	(0.058)	(0.217)	(0.021)
Joyful	0.053	0.167	0.108***
	(0.053)	(0.226)	(0.014)
Angry	-0.013	0.222	-0.087***
	(0.145)	(0.127)	(0.015)
Sad	0.071	-0.192	-0.026**
	(0.079)	(0.213)	(0.009)
Stressed	0.053	-0.202	-0.029
	(0.100)	(0.219)	(0.015)
Fearful	-0.099	-0.191	-0.040**
	(0.071)	(0.236)	(0.015)
Disgusted	0.224	0.403	-0.059***
	(0.136)	(0.183)	(0.015)
Indifferent	-0.046	-0.419	-0.104***
	(0.158)	(0.258)	(0.025)
Colombia			
Optimistic	0.045	0.144*	0.240***
	(0.066)	(0.065)	(0.024)
Joyful	-0.017	0.027	0.078***
	(0.061)	(0.035)	(0.015)
Angry	0.040	0.027	-0.062***
	(0.068)	(0.046)	(0.014)
Sad	0.033	-0.020	-0.057***
	(0.065)	(0.039)	(0.012)
Stressed	0.050	-0.032	-0.032*
	(0.067)	(0.056)	(0.016)
Fearful	0.152	-0.015	-0.053**
	(0.083)	(0.061)	(0.020)
Disgusted	0.083	0.080	-0.014
	(0.076)	(0.041)	(0.011)
Indifferent	-0.226	-0.140	-0.120***
	(0.123)	(0.080)	(0.028)

Note: Robust standard errors in parentheses. *** $p < 0.001$, ** $p < 0.01$, * $p < 0.05$. Each cell corresponds to a different regression using only the restricted sample indicated in the header. Coefficients represent the effect of the confirmation frame on the emotion indicated in the first column compared against the refutation frame. All regressions control for age, sex, educational attainment, employment status, partisan attachment, having had COVID-19, number of doses administered of COVID-19 vaccine, and time spent reading the post.

† This category comprises around or less than 10% of the observations.

5.2 Education and attention

In addition to the evidence on heterogeneity by partisan attachment and vaccination status, Tables S23 through S28 add information on the (lack of) heterogeneity in effects by education, time spent reading the question, and attention level. As shown in these tables, there are no significant patterns across countries that support the cognitive hypothesis. As the effect of the confirmation frame is stable across educational attainment on reactions propensity (Table S23) and emotions generation (Table S24), there is no strong evidence that more educated people are less susceptible to the framing[1]. Moreover, we compare how the confirmation framing effect varies according to how long do respondents take to answer and how they perform in a simple attention check and find that it is stable. The attention check is defined as successfully solving the operation $(2/2)+2$ in an independent question. Tables S25 to S28 show that those respondents who spend more time reading the question of interest and those who successfully pass the attention check are either equally or more susceptible to the confirmation frame than those answering more quickly and the ones who fail to solve the mathematical operation. Together, all these results contradict the cognitive burden hypothesis discussed in the main article.

Table S23 Heterogeneity in effects on reactions in the general specification by educational attainment and country. Difference of means of reactions by confirmation and refutation frame assignment, educational attainment, and country.

Variable	Incomplete secondary (or -)†	Completed secondary	Incomplete college	Completed college	Incomplete graduate (or +)
Argentina					
Engage	0.309***	0.219***	0.167***	0.166***	0.180*
	(0.074)	(0.042)	(0.032)	(0.031)	(0.075)
Like	0.151**	0.175***	0.146***	0.170***	0.153*
	(0.055)	(0.034)	(0.026)	(0.026)	(0.061)
Share	0.142*	0.050	0.040	0.021	0.022
	(0.062)	(0.028)	(0.022)	(0.020)	(0.052)
Comment	0.046	0.025	-0.002	0.000	0.022
	(0.035)	(0.021)	(0.015)	(0.014)	(0.034)
Brazil					
Engage	0.011	0.158***	0.137*	0.089	0.196***
	(0.083)	(0.045)	(0.064)	(0.047)	(0.052)

[1] The Argentina sample is the only one showing a slightly higher effect on engagement for the lowest education category, but this is not significantly different than the effect in any other group.

Like	0.065	0.151***	0.155*	0.077	0.176***
	(0.071)	(0.041)	(0.060)	(0.044)	(0.051)
Share	-0.019	-0.013	0.026	-0.000	0.004
	(0.056)	(0.033)	(0.048)	(0.035)	(0.038)
Comment	-0.039	-0.031	-0.008	0.043	0.002
	(0.042)	(0.032)	(0.040)	(0.028)	(0.030)
Chile					
Engage	0.243	0.157**	0.096	0.179***	0.163*
	(0.309)	(0.052)	(0.051)	(0.034)	(0.071)
Like	-0.062	0.146***	0.204***	0.178***	0.200**
	(0.223)	(0.040)	(0.037)	(0.028)	(0.065)
Share	0.241	-0.002	-0.029	0.040	-0.046
	(0.356)	(0.040)	(0.040)	(0.024)	(0.047)
Comment	0.021	0.007	-0.035	0.020	-0.000
	(0.085)	(0.027)	(0.026)	(0.012)	(0.029)
Colombia					
Engage	0.327*	0.110*	0.154**	0.161***	0.117
	(0.147)	(0.053)	(0.050)	(0.039)	(0.078)
Like	0.147	0.159***	0.100*	0.098**	0.187**
	(0.110)	(0.042)	(0.043)	(0.031)	(0.067)
Share	0.137	-0.032	0.055	0.065*	-0.017
	(0.157)	(0.041)	(0.036)	(0.031)	(0.071)
Comment	0.086	0.022	0.028	0.021	0.019
	(0.078)	(0.030)	(0.027)	(0.018)	(0.028)

Note: Robust standard errors in parentheses. *** $p < 0.001$, ** $p < 0.01$, * $p < 0.05$. Each cell corresponds to a different regression using only the restricted sample indicated in the header. Coefficients represent the effect of the confirmation frame on the reaction indicated in the first column compared against the refutation frame. All regressions control for age, sex, educational attainment, employment status, partisan attachment, having had COVID-19, number of doses administered of COVID-19 vaccine, and time spent reading the post.

† This category comprises less than 5% of the observations.

Table S24 Heterogeneity in effects on emotions generation in the general specification by educational attainment and country. Difference of means of emotions generation by confirmation and refutation frame assignment, educational attainment, and country.

Variable	Incomplete secondary (or -)†	Completed secondary	Incomplete college	Completed college	Incomplete graduate (or +)
Argentina					
Optimistic	0.339***	0.231***	0.321***	0.280***	0.159*
	(0.062)	(0.034)	(0.027)	(0.026)	(0.061)

Joyful	0.062*	0.089***	0.099***	0.053***	0.191***
	(0.028)	(0.020)	(0.016)	(0.016)	(0.054)
Angry	-0.026	-0.110***	-0.141***	-0.118***	-0.116**
	(0.040)	(0.027)	(0.023)	(0.021)	(0.044)
Sad	-0.076*	-0.033	-0.031*	-0.032*	-0.044
	(0.034)	(0.021)	(0.013)	(0.014)	(0.026)
Stressed	-0.036	-0.059	-0.110***	-0.066***	-0.004
	(0.061)	(0.031)	(0.022)	(0.017)	(0.035)
Fearful	-0.047	-0.006	-0.003	-0.032**	0.008
	(0.035)	(0.022)	(0.012)	(0.012)	(0.009)
Disgusted	-0.131*	-0.068*	-0.126***	-0.135***	-0.203**
	(0.061)	(0.031)	(0.023)	(0.024)	(0.062)
Indifferent	-0.095	-0.025	-0.052	0.000	0.010
	(0.086)	(0.046)	(0.036)	(0.034)	(0.084)
Brazil					
Optimistic	0.139	0.182***	0.269***	0.289***	0.384***
	(0.072)	(0.040)	(0.060)	(0.041)	(0.047)
Joyful	0.092	0.060	0.190***	0.111**	0.123**
	(0.053)	(0.032)	(0.054)	(0.034)	(0.042)
Angry	-0.049	-0.081**	-0.096*	-0.066*	-0.123***
	(0.028)	(0.025)	(0.039)	(0.026)	(0.031)
Sad	-0.116*	-0.092***	-0.133***	-0.074**	-0.118***
	(0.055)	(0.025)	(0.040)	(0.025)	(0.030)
Stressed	-0.005	-0.028	-0.100**	-0.053*	-0.060*
	(0.024)	(0.020)	(0.033)	(0.026)	(0.030)
Fearful	-0.099	-0.012	-0.056	-0.047	-0.093**
	(0.058)	(0.024)	(0.038)	(0.024)	(0.032)
Disgusted	-0.064	-0.088***	-0.178***	-0.076**	-0.171***
	(0.039)	(0.026)	(0.039)	(0.026)	(0.035)
Indifferent	0.038	-0.029	0.015	-0.066	-0.061
	(0.086)	(0.044)	(0.062)	(0.044)	(0.046)
Chile					
Optimistic	0.497*	0.165***	0.161***	0.317***	0.313***
	(0.178)	(0.045)	(0.042)	(0.030)	(0.065)
Joyful	0.100	0.069**	0.096***	0.123***	0.183***
	(0.104)	(0.026)	(0.028)	(0.021)	(0.052)
Angry	-0.177	-0.080*	-0.086*	-0.080***	-0.086*
	(0.264)	(0.034)	(0.035)	(0.021)	(0.041)
Sad	0.000	-0.026	-0.002	-0.041**	0.000
		(0.020)	(0.022)	(0.013)	(0.021)

Stressed	-0.329	-0.006	-0.028	-0.018	-0.046
	(0.238)	(0.035)	(0.037)	(0.022)	(0.034)
Fearful	0.140	-0.087*	-0.040	-0.042*	-0.019
	(0.184)	(0.039)	(0.033)	(0.020)	(0.038)
Disgusted	0.002	0.021	-0.032	-0.084***	-0.092*
	(0.154)	(0.031)	(0.038)	(0.024)	(0.038)
Indifferent	-0.047	-0.023	-0.077	-0.141***	-0.154*
	(0.391)	(0.056)	(0.054)	(0.036)	(0.074)
Colombia					
Optimistic	0.265*	0.225***	0.284***	0.201***	0.168*
	(0.121)	(0.048)	(0.044)	(0.035)	(0.077)
Joyful	0.088	0.074*	0.068*	0.079***	0.024
	(0.073)	(0.030)	(0.028)	(0.020)	(0.043)
Angry	0.076	-0.043	-0.033	-0.069**	-0.060
	(0.107)	(0.027)	(0.023)	(0.022)	(0.046)
Sad	0.046	-0.039	-0.075**	-0.028	-0.091*
	(0.080)	(0.030)	(0.024)	(0.016)	(0.036)
Stressed	0.191	0.006	-0.044	-0.047*	-0.085
	(0.120)	(0.037)	(0.029)	(0.022)	(0.057)
Fearful	0.130	-0.047	-0.100**	0.004	-0.129*
	(0.113)	(0.046)	(0.036)	(0.029)	(0.053)
Disgusted	0.060	0.035	-0.018	-0.011	-0.047
	(0.075)	(0.026)	(0.017)	(0.017)	(0.030)
Indifferent	-0.505***	-0.168**	-0.122*	-0.122**	0.062
	(0.128)	(0.052)	(0.053)	(0.040)	(0.088)

Note: Robust standard errors in parentheses. *** p < 0.001, ** p < 0.01, * p < 0.05. Each cell corresponds to a different regression using only the restricted sample indicated in the header. Coefficients represent the effect of the confirmation frame on the emotion indicated in the first column compared against the refutation frame. All regressions control for age, sex, educational attainment, employment status, partisan attachment, having had COVID-19, number of doses administered of COVID-19 vaccine, and time spent reading the post.

† This category comprises less than 5% of the observations.

Table S25 Heterogeneity in effects on reactions in the general specification by quintile of time spent reading the question and country. Difference of means of reactions by confirmation and refutation frame assignment, quintile of time spent reading, and country.

Variable	First quintile	Third quintile	Fifth quintile	Fifth v. first quintile
Argentina				
Engage	0.150***	0.139***	0.247***	0.102
	(0.040)	(0.042)	(0.043)	(0.057)

Like	0.141***	0.151***	0.217***	0.089
	(0.034)	(0.034)	(0.034)	(0.047)
Share	0.024	0.018	0.080*	0.053
	(0.025)	(0.029)	(0.031)	(0.039)
Comment	-0.011	0.013	-0.003	0.002
	(0.016)	(0.016)	(0.025)	(0.029)
Brazil				
Engage	0.089	0.076	0.150**	0.072
	(0.059)	(0.053)	(0.051)	(0.077)
Like	0.060	0.099	0.091	0.033
	(0.054)	(0.051)	(0.047)	(0.070)
Share	0.014	-0.066	0.009	0.005
	(0.045)	(0.040)	(0.039)	(0.058)
Comment	-0.021	-0.016	0.043	0.062
	(0.028)	(0.027)	(0.040)	(0.049)
Chile				
Engage	0.235***	0.142**	0.175***	-0.103
	(0.055)	(0.052)	(0.052)	(0.074)
Like	0.213***	0.191***	0.120**	-0.117
	(0.048)	(0.044)	(0.040)	(0.060)
Share	0.015	0.039	0.030	-0.003
	(0.037)	(0.041)	(0.042)	(0.054)
Comment	0.006	0.017	0.066*	0.051
	(0.020)	(0.019)	(0.027)	(0.033)
Colombia				
Engage	0.201**	0.062	0.119*	-0.081
	(0.067)	(0.057)	(0.052)	(0.081)
Like	0.189***	0.083	0.080	-0.086
	(0.057)	(0.046)	(0.041)	(0.067)
Share	0.047	-0.021	0.076	0.027
	(0.051)	(0.043)	(0.043)	(0.063)
Comment	-0.019	0.010	0.025	0.020
	(0.029)	(0.024)	(0.031)	(0.042)

Note: Robust standard errors in parentheses. *** $p < 0.001$, ** $p < 0.01$, * $p < 0.05$. Each cell in the first three columns corresponds to a different regression using only the restricted sample indicated in the header. Coefficients represent the effect of the confirmation frame on the reaction indicated in the first column compared against the refutation frame. The last column present the difference between coefficients in the third and first columns from the interaction of the treatment with the categories indicated. All regressions control for age, sex, educational attainment, employment status, partisan attachment, having had COVID-19, number of doses administered of COVID-19 vaccine, and time spent reading the post.

Table S26 Heterogeneity in effects on emotions generation in the general specification by quintile of time spent reading the question and country. Difference of means of emotions generation by confirmation and refutation frame assignment, quintile of time spent reading, and country.

Variable	First quintile	Third quintile	Fifth quintile	Fifth v. first quintile
Argentina				
Optimistic	0.206***	0.279***	0.371***	0.157**
	(0.032)	(0.037)	(0.036)	(0.048)
Joyful	0.084***	0.075***	0.062**	-0.017
	(0.021)	(0.021)	(0.020)	(0.029)
Angry	-0.111***	-0.099***	-0.121***	-0.010
	(0.028)	(0.027)	(0.031)	(0.040)
Sad	-0.032	-0.028	-0.030	-0.003
	(0.019)	(0.015)	(0.020)	(0.027)
Stressed	-0.059*	-0.125***	-0.090***	-0.030
	(0.026)	(0.032)	(0.027)	(0.036)
Fearful	-0.016	-0.020	-0.017	0.002
	(0.016)	(0.020)	(0.019)	(0.024)
Disgusted	-0.120***	-0.073*	-0.155***	-0.032
	(0.033)	(0.029)	(0.032)	(0.045)
Indifferent	0.021	-0.034	-0.061	-0.085
	(0.047)	(0.047)	(0.044)	(0.064)
Brazil				
Optimistic	0.182***	0.289***	0.226***	0.056
	(0.049)	(0.048)	(0.048)	(0.067)
Joyful	0.067	0.079	0.132***	0.070
	(0.038)	(0.043)	(0.037)	(0.053)
Angry	-0.080**	-0.063*	-0.077**	0.015
	(0.030)	(0.031)	(0.029)	(0.042)
Sad	-0.095**	-0.065*	-0.111***	-0.023
	(0.033)	(0.025)	(0.033)	(0.047)
Stressed	-0.083*	-0.027	-0.015	0.062
	(0.036)	(0.026)	(0.025)	(0.042)
Fearful	-0.053	-0.019	-0.054	0.012
	(0.031)	(0.026)	(0.036)	(0.047)
Disgusted	-0.141***	-0.131***	-0.137***	0.005
	(0.033)	(0.031)	(0.033)	(0.046)
Indifferent	0.039	-0.036	-0.004	-0.070
	(0.056)	(0.050)	(0.048)	(0.073)

Chile				
Optimistic	0.257***	0.271***	0.244***	-0.031
	(0.047)	(0.048)	(0.047)	(0.064)
Joyful	0.135***	0.126***	0.157***	0.012
	(0.033)	(0.031)	(0.037)	(0.048)
Angry	-0.065	-0.053	-0.108**	-0.036
	(0.033)	(0.033)	(0.033)	(0.047)
Sad	-0.020	-0.014	-0.024	-0.005
	(0.015)	(0.019)	(0.019)	(0.024)
Stressed	-0.086*	0.022	0.011	0.080
	(0.036)	(0.030)	(0.034)	(0.049)
Fearful	-0.060*	-0.014	-0.029	0.013
	(0.030)	(0.032)	(0.037)	(0.046)
Disgusted	-0.051	-0.026	-0.027	0.011
	(0.034)	(0.034)	(0.036)	(0.049)
Indifferent	-0.113	-0.157**	-0.138**	0.005
	(0.061)	(0.057)	(0.053)	(0.079)
Colombia				
Optimistic	0.211***	0.204***	0.174***	-0.019
	(0.059)	(0.052)	(0.047)	(0.071)
Joyful	0.049	0.029	0.067*	0.008
	(0.034)	(0.025)	(0.030)	(0.044)
Angry	-0.048	-0.066*	-0.047	-0.006
	(0.029)	(0.028)	(0.030)	(0.041)
Sad	0.002	-0.073**	-0.047	-0.043
	(0.025)	(0.024)	(0.030)	(0.041)
Stressed	0.048	-0.037	-0.029	-0.066
	(0.044)	(0.034)	(0.034)	(0.053)
Fearful	-0.025	-0.069	-0.026	0.006
	(0.045)	(0.039)	(0.043)	(0.060)
Disgusted	0.001	-0.033	0.040	0.057
	(0.019)	(0.028)	(0.022)	(0.030)
Indifferent	-0.182**	0.009	-0.149**	0.019
	(0.067)	(0.059)	(0.052)	(0.081)

Note: Robust standard errors in parentheses. *** $p < 0.001$, ** $p < 0.01$, * $p < 0.05$. Each cell in the first three columns corresponds to a different regression using only the restricted sample indicated in the header. Coefficients represent the effect of the confirmation frame on the emotion indicated in the first column compared against the refutation frame. The last column present the difference between coefficients in the third and first columns from the interaction of the treatment with the categories indicated. All regressions control for age,

sex, educational attainment, employment status, partisan attachment, having had COVID-19, number of doses administered of COVID-19 vaccine, and time spent reading the post.

Table S27 Heterogeneity in effects on reactions in the general specification by fulfillment of attention check and country. Difference of means of reactions by confirmation and refutation frame assignment, fulfillment of attention check, and country.

Variable	Inattentive	Attentive	Difference
Brazil			
Engage	0.112*	0.138***	0.020
	(0.045)	(0.028)	(0.053)
Like	0.104*	0.134***	0.026
	(0.041)	(0.026)	(0.049)
Share	-0.001	0.001	0.002
	(0.033)	(0.021)	(0.039)
Comment	-0.008	0.004	0.008
	(0.030)	(0.017)	(0.035)
Chile			
Engage	0.174***	0.145***	-0.021
	(0.044)	(0.027)	(0.051)
Like	0.157***	0.177***	0.031
	(0.034)	(0.022)	(0.040)
Share	0.015	0.006	-0.008
	(0.032)	(0.020)	(0.037)
Comment	-0.008	0.010	0.014
	(0.022)	(0.011)	(0.024)
Colombia			
Engage	0.189***	0.141***	-0.024
	(0.049)	(0.028)	(0.056)
Like	0.129**	0.114***	-0.013
	(0.039)	(0.023)	(0.045)
Share	0.020	0.045*	0.034
	(0.041)	(0.022)	(0.045)
Comment	0.098***	0.006	-0.084**
	(0.028)	(0.014)	(0.031)

Note: Robust standard errors in parentheses. *** $p < 0.001$, ** $p < 0.01$, * $p < 0.05$. Each cell in the first two columns corresponds to a different regression using only the restricted sample indicated in the header. Coefficients represent the effect of the confirmation frame on the reaction indicated in the first column compared against the refutation frame. The last column present the difference between coefficients in the second and first columns from the interaction of the treatment with the categories indicated. All regressions control for age, sex, educational attainment, employment status, partisan attachment, having had COVID-19, number of doses administered of COVID-19 vaccine, and time spent reading the post.

Table S28 Heterogeneity in effects on emotions generation in the general specification by fulfillment of attention check and country. Difference of means of emotions generation by confirmation and refutation frame assignment, fulfillment of attention check, and country.

Variable	Inattentive	Attentive	Difference
Brazil			
Optimistic	0.187***	0.293***	0.114*
	(0.040)	(0.026)	(0.047)
Joyful	0.061*	0.126***	0.068
	(0.030)	(0.022)	(0.037)
Angry	-0.097***	-0.079***	0.014
	(0.023)	(0.017)	(0.028)
Sad	-0.108***	-0.098***	0.006
	(0.028)	(0.015)	(0.031)
Stressed	-0.011	-0.068***	-0.057*
	(0.021)	(0.015)	(0.025)
Fearful	-0.032	-0.057***	-0.023
	(0.025)	(0.016)	(0.030)
Disgusted	-0.106***	-0.117***	-0.014
	(0.025)	(0.018)	(0.030)
Indifferent	-0.016	-0.035	-0.025
	(0.043)	(0.027)	(0.051)
Chile			
Optimistic	0.265***	0.243***	-0.013
	(0.037)	(0.024)	(0.043)
Joyful	0.100***	0.107***	0.008
	(0.025)	(0.016)	(0.029)
Angry	-0.089**	-0.081***	0.006
	(0.030)	(0.017)	(0.035)
Sad	-0.020	-0.023*	-0.002
	(0.017)	(0.010)	(0.020)
Stressed	-0.045	-0.017	0.024
	(0.030)	(0.017)	(0.035)
Fearful	-0.080**	-0.029	0.054
	(0.031)	(0.017)	(0.034)
Disgusted	-0.013	-0.061***	-0.057
	(0.033)	(0.017)	(0.037)
Indifferent	-0.097*	-0.103***	-0.013
	(0.049)	(0.029)	(0.056)
Colombia			

Optimistic	0.282***	0.209***	-0.069
	(0.044)	(0.025)	(0.050)
Joyful	0.073**	0.068***	-0.010
	(0.026)	(0.015)	(0.029)
Angry	-0.017	-0.059***	-0.037
	(0.022)	(0.015)	(0.028)
Sad	-0.077**	-0.038**	0.044
	(0.025)	(0.013)	(0.028)
Stressed	-0.007	-0.037*	-0.027
	(0.031)	(0.017)	(0.036)
Fearful	-0.026	-0.041*	-0.003
	(0.042)	(0.020)	(0.046)
Disgusted	0.006	-0.003	-0.007
	(0.020)	(0.013)	(0.025)
Indifferent	-0.155**	-0.121***	0.038
	(0.051)	(0.029)	(0.057)

Note: Robust standard errors in parentheses. *** $p < 0.001$, ** $p < 0.01$, * $p < 0.05$. Each cell in the first two columns corresponds to a different regression using only the restricted sample indicated in the header. Coefficients represent the effect of the confirmation frame on the emotion indicated in the first column compared against the refutation frame. The last column present the difference between coefficients in the second and first columns from the interaction of the treatment with the categories indicated. All regressions control for age, sex, educational attainment, employment status, partisan attachment, having had COVID-19, number of doses administered of COVID-19 vaccine, and time spent reading the post.

6 Placebo experiment

In addition to the vaccine framings, a placebo experiment was conducted using a picture of a dog and varying only the first sentence of the statement: “It is TRUE” or ”It is FALSE”. The second sentence was common to both framings and read “Study shows that dogs do not really know what you are saying to them” (see Figure S6). Tables S29 to S31 present the results for the placebo framings for Brazil, Chile, and Colombia, respectively. In all cases, albeit attenuated, there is still a positive effect of the confirmation frame on engagement through likes, but all the other dependent variables are found to be framing invariant. This result, together with the heterogeneity by partisan attachment presented in section 5.1, can be interpreted as showing that framing effects depend on how salient the issue is to the reader and how it reinforces or contradicts an ideological prior rather than a non-ideological fact.

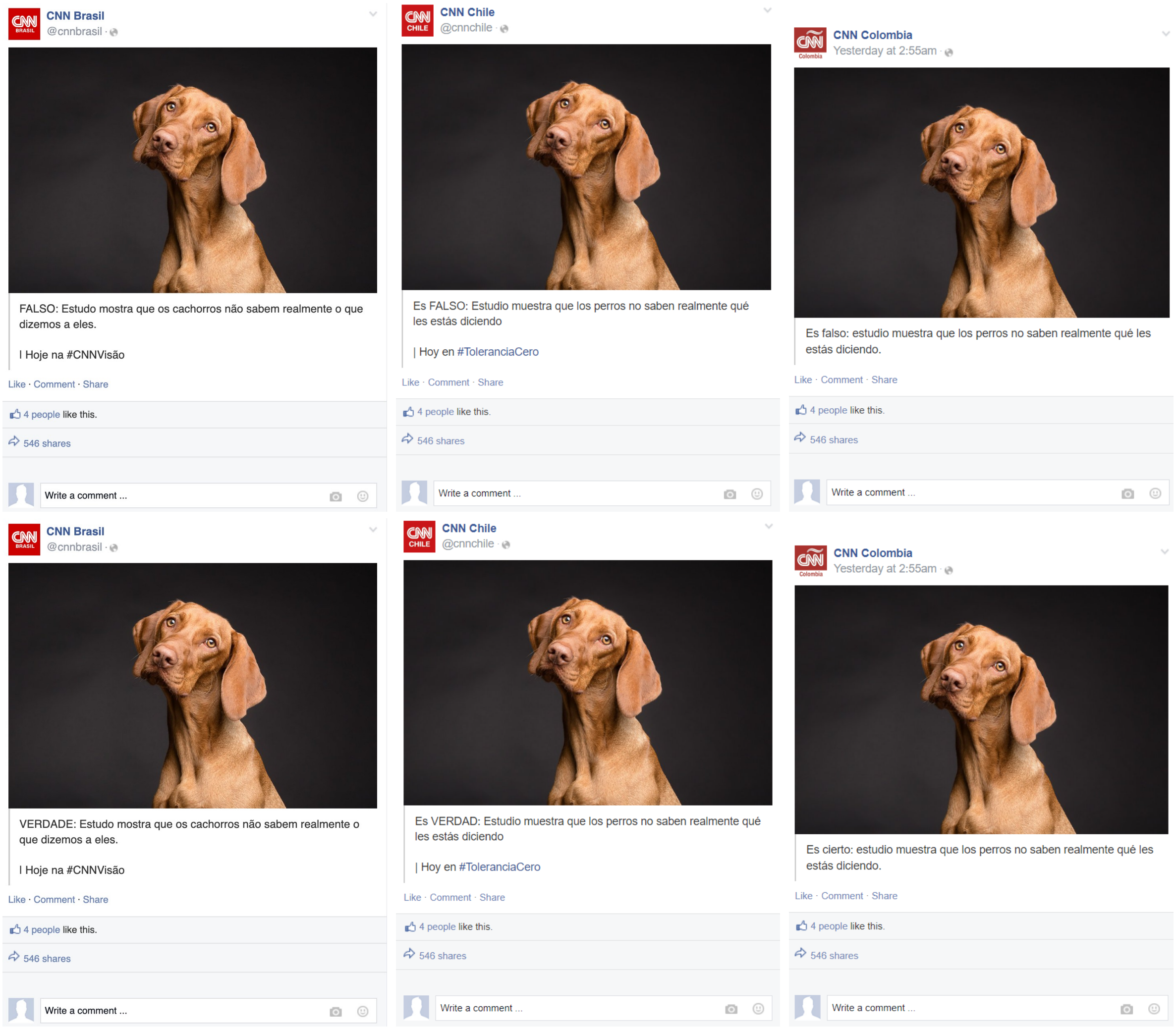

Figure S6 Images of the *confirmation* and *refutation* placebo treatments used in Brazil, Chile, and Colombia. The confirmation and refutation frames are semantically equivalent and intended to be equivalent in their cognitive accessibility and their valence charge, changing only the word "True" for "False". The texts read "It is [TRUE/FALSE]. Study shows that dogs do not really know what you are saying to them". All four treatments are factually correct and conform to the design used by our partner organization in Argentina, *Chequeado*.

Table S29 Difference of means between the confirmation and refutation frames in the placebo (dog) experiment in the Brazil survey.

Variable	Refutation frame	Confirmation frame	Simple difference	Difference with controls
Reactions				
Engage	0.354	0.431	0.077*	0.066
	(0.025)	(0.025)	(0.036)	(0.036)
Like	0.248	0.317	0.069*	0.063
	(0.023)	(0.024)	(0.033)	(0.033)
Share	0.076	0.086	0.009	0.007
	(0.014)	(0.014)	(0.020)	(0.020)
Comment	0.120	0.114	-0.006	-0.012
	(0.017)	(0.016)	(0.023)	(0.024)
Emotions				
Optimistic	0.087	0.086	-0.001	-0.007
	(0.015)	(0.014)	(0.021)	(0.021)
Joyful	0.082	0.068	-0.014	-0.016
	(0.014)	(0.013)	(0.019)	(0.019)
Angry	0.054	0.042	-0.013	-0.012
	(0.012)	(0.010)	(0.016)	(0.016)
Sad	0.125	0.096	-0.029	-0.028
	(0.017)	(0.015)	(0.023)	(0.023)
Stressed	0.030	0.031	0.001	-0.003
	(0.009)	(0.009)	(0.013)	(0.013)
Fearful	0.027	0.010	-0.017	-0.019
	(0.009)	(0.005)	(0.010)	(0.010)
Disgusted	0.068	0.081	0.012	0.009
	(0.013)	(0.014)	(0.019)	(0.019)
Indifferent	0.629	0.642	0.012	0.022
	(0.025)	(0.024)	(0.035)	(0.035)

Note: Robust standard errors in parentheses. *** $p < 0.001$, ** $p < 0.01$, * $p < 0.05$. The first two columns represent average values for each reaction and emotion in response to the refutation and confirmation frames respectively in the placebo experiment. The third column presents the difference between the confirmation and refutation frame without controls. Differences in the fourth column are estimated controlling for age, sex, educational attainment, employment status, partisan attachment, having had COVID-19, number of doses administered of COVID-19 vaccine, and time spent reading the post. Significance levels are derived from linear regression models.

Table S30 Difference of means between the confirmation and refutation frames in the placebo (dog) experiment in the Chile survey.

Variable	Refutation frame	Confirmation frame	Simple difference	Difference with controls
Reactions				
Engage	0.354	0.434	0.080*	0.084*
	(0.025)	(0.025)	(0.035)	(0.036)
Like	0.192	0.267	0.075*	0.071*
	(0.021)	(0.022)	(0.030)	(0.031)
Share	0.109	0.125	0.016	0.021
	(0.016)	(0.017)	(0.023)	(0.023)
Comment	0.148	0.142	-0.005	0.007
	(0.019)	(0.017)	(0.026)	(0.026)
Emotions				
Optimistic	0.089	0.127	0.038	0.045*
	(0.015)	(0.017)	(0.022)	(0.023)
Joyful	0.081	0.090	0.009	0.012
	(0.014)	(0.014)	(0.020)	(0.020)
Angry	0.109	0.082	-0.026	-0.025
	(0.016)	(0.014)	(0.021)	(0.022)
Sad	0.134	0.150	0.016	0.009
	(0.018)	(0.018)	(0.025)	(0.026)
Stressed	0.036	0.022	-0.014	-0.013
	(0.010)	(0.007)	(0.012)	(0.013)
Fearful	0.019	0.015	-0.005	-0.004
	(0.007)	(0.006)	(0.009)	(0.010)
Disgusted	0.031	0.025	-0.006	-0.005
	(0.009)	(0.008)	(0.012)	(0.012)
Indifferent	0.610	0.586	-0.024	-0.021
	(0.026)	(0.025)	(0.036)	(0.036)

Note: Robust standard errors in parentheses. *** $p < 0.001$, ** $p < 0.01$, * $p < 0.05$. The first two columns represent average values for each reaction and emotion in response to the refutation and confirmation frames respectively in the placebo experiment. The third column presents the difference between the confirmation and refutation frame without controls. Differences in the fourth column are estimated controlling for age, sex, educational attainment, employment status, partisan attachment, having had COVID-19, number of doses administered of COVID-19 vaccine, and time spent reading the post. Significance levels are derived from linear regression models.

Table S31 Difference of means between the confirmation and refutation frames in the placebo (dog) experiment in the Colombia survey.

Variable	Refutation frame	Confirmation frame	Simple difference	Difference with controls
Reactions				
Engage	0.440	0.529	0.089*	0.087*
	(0.025)	(0.025)	(0.035)	(0.035)
Like	0.246	0.267	0.021	0.020
	(0.021)	(0.022)	(0.030)	(0.031)
Share	0.123	0.192	0.069**	0.067**
	(0.016)	(0.019)	(0.025)	(0.025)
Comment	0.162	0.190	0.028	0.028
	(0.018)	(0.019)	(0.027)	(0.027)
Emotions				
Optimistic	0.118	0.156	0.038	0.038
	(0.016)	(0.018)	(0.024)	(0.024)
Joyful	0.106	0.123	0.017	0.016
	(0.015)	(0.016)	(0.022)	(0.022)
Angry	0.076	0.125	0.049*	0.048*
	(0.013)	(0.016)	(0.021)	(0.021)
Sad	0.147	0.147	-0.001	0.004
	(0.018)	(0.017)	(0.025)	(0.025)
Stressed	0.015	0.012	-0.003	-0.000
	(0.006)	(0.005)	(0.008)	(0.007)
Fearful	0.017	0.026	0.009	0.015
	(0.006)	(0.008)	(0.010)	(0.010)
Disgusted	0.022	0.017	-0.005	-0.008
	(0.007)	(0.006)	(0.010)	(0.010)
Indifferent	0.592	0.536	-0.056	-0.057
	(0.024)	(0.024)	(0.035)	(0.034)

Note: Robust standard errors in parentheses. *** $p < 0.001$, ** $p < 0.01$, * $p < 0.05$. The first two columns represent average values for each reaction and emotion in response to the refutation and confirmation frames respectively in the placebo experiment. The third column presents the difference between the confirmation and refutation frame without controls. Differences in the fourth column are estimated controlling for age, sex, educational attainment, employment status, partisan attachment, having had COVID-19, number of doses administered of COVID-19 vaccine, and time spent reading the post. Significance levels are derived from linear regression models.

7 IRB Approval

We received IRB approval for this project's four surveys and other data collections. The initial application for the Argentina Survey is [1825785-1] and [1825785-3], COVID-19, Trust, and Misinformation. We then requested separate approvals for surveys in Chile [1825785-6], Brazil [1825785-7], and Colombia [1825785-8]. As part of this project, we also requested approvals for the collection of social media data in [1825785-2], [1825785-4], [1825785-5], and [1825785-9]. Below we present the four IRB letters of approval, one for each survey.

UNIVERSITY OF MARYLAND
INSTITUTIONAL REVIEW BOARD

1204 Marie Mount Hall
College Park, MD 20742-5125
TEL 301.405.4212
FAX 301.314.1475
irb@umd.edu
www.umresearch.umd.edu/IRB

DATE:	October 27, 2021
TO:	Ernesto Calvo, PhD
FROM:	University of Maryland College Park (UMCP) IRB
PROJECT TITLE:	[1825785-1] COVID-19, Trust, and Misinformation
SUBMISSION TYPE:	New Project
ACTION:	APPROVED
APPROVAL DATE:	October 27, 2021
REVIEW TYPE:	Expedited Review
REVIEW CATEGORY:	Expedited review category # *7, Waiver of written consent, 45CFR46.116(f)(3), Use of deception, 45CFR46.116(f)(2).*

Thank you for your submission of New Project materials for this project. The University of Maryland College Park (UMCP) IRB has APPROVED your submission. This approval is based on an appropriate risk/benefit ratio and a project design wherein the risks have been minimized. All research must be conducted in accordance with this approved submission.

Prior to final approval of this project scientific review was completed by the IRB Member reviewer.

This submission has received Expedited Review based on the applicable federal regulations.

This project has been determined to be a MINIMAL RISK project.

Please remember that informed consent is a process beginning with a description of the project and insurance of participant understanding followed by a signed consent form. Informed consent must continue throughout the project via a dialogue between the researcher and research participant. Unless a consent waiver or alteration has been approved, Federal regulations require that each participant receives a copy of the consent document.

Please note that any revision to previously approved materials must be approved by this committee prior to initiation. Please use the appropriate Amendment forms for this procedure.

All UNANTICIPATED PROBLEMS involving risks to subjects or others (UPIRSOs) and SERIOUS and UNEXPECTED adverse events must be reported promptly to this office. Please use the appropriate reporting forms for this procedure. All FDA and sponsor reporting requirements should also be followed. All NON-COMPLIANCE issues or COMPLAINTS regarding this project must be reported promptly to this office.

Please note that all research records must be retained for a minimum of seven years after the completion of the project.

If you have any questions, please contact the IRB Office at 301-405-4212 or irb@umd.edu. Please include your project title and reference number in all correspondence with this committee.

Generated on IRBNet

This letter has been electronically signed in accordance with all applicable regulations, and a copy is retained within University of Maryland College Park (UMCP) IRB's records.

Generated on IRBNet

UNIVERSITY OF MARYLAND
INSTITUTIONAL REVIEW BOARD

1204 Marie Mount Hall
College Park, MD 20742-5125
TEL 301.405.4212
FAX 301.314.1475
irb@umd.edu
www.umresearch.umd.edu/IRB

DATE:	February 3, 2022
TO:	Ernesto Calvo, PhD
FROM:	University of Maryland College Park (UMCP) IRB
PROJECT TITLE:	[1825785-3] COVID-19, Trust, and Misinformation
SUBMISSION TYPE:	Amendment/Modification
ACTION:	APPROVED
APPROVAL DATE:	February 3, 2022
REVIEW TYPE:	Expedited Review
REVIEW CATEGORY:	Expedited review category # *7, Waiver of consent (deception), 45CFR46.116(f)(3).*

Thank you for your submission of Amendment/Modification materials for this project. The University of Maryland College Park (UMCP) IRB has APPROVED your submission. This approval is based on an appropriate risk/benefit ratio and a project design wherein the risks have been minimized. All research must be conducted in accordance with this approved submission.

Prior to final approval of this project scientific review was completed by the IRB Member reviewer.

This submission has received Expedited Review based on the applicable federal regulations.

This project has been determined to be a MINIMAL RISK project.

Please remember that informed consent is a process beginning with a description of the project and insurance of participant understanding followed by a signed consent form. Informed consent must continue throughout the project via a dialogue between the researcher and research participant. Unless a consent waiver or alteration has been approved, Federal regulations require that each participant receives a copy of the consent document.

Please note that any revision to previously approved materials must be approved by this committee prior to initiation. Please use the appropriate Amendment forms for this procedure.

All UNANTICIPATED PROBLEMS involving risks to subjects or others (UPIRSOs) and SERIOUS and UNEXPECTED adverse events must be reported promptly to this office. Please use the appropriate reporting forms for this procedure. All FDA and sponsor reporting requirements should also be followed. All NON-COMPLIANCE issues or COMPLAINTS regarding this project must be reported promptly to this office.

Please note that all research records must be retained for a minimum of seven years after the completion of the project.

If you have any questions, please contact the IRB Office at 301-405-4212 or irb@umd.edu. Please include your project title and reference number in all correspondence with this committee.

Generated on IRBNet

This letter has been electronically signed in accordance with all applicable regulations, and a copy is retained within University of Maryland College Park (UMCP) IRB's records.

Generated on IRBNet

UNIVERSITY OF MARYLAND
INSTITUTIONAL REVIEW BOARD

1204 Marie Mount Hall
College Park, MD 20742-5125
TEL 301.405.4212
FAX 301.314.1475
irb@umd.edu
www.umresearch.umd.edu/IRB

DATE:	October 19, 2022
TO:	Ernesto Calvo, PhD
FROM:	University of Maryland College Park (UMCP) IRB
PROJECT TITLE:	[1825785-6] COVID-19, Trust, and Misinformation
SUBMISSION TYPE:	Amendment/Modification
ACTION:	APPROVED
APPROVAL DATE:	October 19, 2022
REVIEW TYPE:	Expedited Review
REVIEW CATEGORY:	Expedited review category # *7, Waiver of consent (deception) under 45CFR46.116(f)(3).*

Thank you for your submission of Amendment/Modification materials for this project. The University of Maryland College Park (UMCP) IRB has APPROVED your submission. This approval is based on an appropriate risk/benefit ratio and a project design wherein the risks have been minimized. All research must be conducted in accordance with this approved submission.

Prior to final approval of this project scientific review was completed by the IRB Member reviewer.

This submission has received Expedited Review based on the applicable federal regulations.

This project has been determined to be a MINIMAL RISK project.

Please remember that informed consent is a process beginning with a description of the project and insurance of participant understanding followed by a signed consent form. Informed consent must continue throughout the project via a dialogue between the researcher and research participant. Unless a consent waiver or alteration has been approved, Federal regulations require that each participant receives a copy of the consent document.

Please note that any revision to previously approved materials must be approved by this committee prior to initiation. Please use the appropriate Amendment forms for this procedure.

All UNANTICIPATED PROBLEMS involving risks to subjects or others (UPIRSOs) and SERIOUS and UNEXPECTED adverse events must be reported promptly to this office. Please use the appropriate reporting forms for this procedure. All FDA and sponsor reporting requirements should also be followed. All NON-COMPLIANCE issues or COMPLAINTS regarding this project must be reported promptly to this office.

Please note that all research records must be retained for a minimum of seven years after the completion of the project.

If you have any questions, please contact the IRB Office at 301-405-4212 or irb@umd.edu. Please include your project title and reference number in all correspondence with this committee.

Generated on IRBNet

This letter has been electronically signed in accordance with all applicable regulations, and a copy is retained within University of Maryland College Park (UMCP) IRB's records.

Generated on IRBNet

UNIVERSITY OF MARYLAND
INSTITUTIONAL REVIEW BOARD

1204 Marie Mount Hall
College Park, MD 20742-5125
TEL 301.405.4212
FAX 301.314.1475
irb@umd.edu
www.umresearch.umd.edu/IRB

DATE:	November 28, 2022
TO:	Ernesto Calvo, PhD
FROM:	University of Maryland College Park (UMCP) IRB
PROJECT TITLE:	[1825785-7] COVID-19, Trust, and Misinformation
SUBMISSION TYPE:	Amendment/Modification
ACTION:	APPROVED
APPROVAL DATE:	November 28, 2022
REVIEW TYPE:	Expedited Review
REVIEW CATEGORY:	Expedited review category # 7, *Waiver of consent (deception), 45CFR46,116(f)(2), Waiver of consent documentation, 45CFR46,117(c).*

Thank you for your submission of Amendment/Modification materials for this project. The University of Maryland College Park (UMCP) IRB has APPROVED your submission. This approval is based on an appropriate risk/benefit ratio and a project design wherein the risks have been minimized. All research must be conducted in accordance with this approved submission.

Prior to final approval of this project scientific review was completed by the IRB Member reviewer.

This submission has received Expedited Review based on the applicable federal regulations.

This project has been determined to be a MINIMAL RISK project.

Please remember that informed consent is a process beginning with a description of the project and insurance of participant understanding followed by a signed consent form. Informed consent must continue throughout the project via a dialogue between the researcher and research participant. Unless a consent waiver or alteration has been approved, Federal regulations require that each participant receives a copy of the consent document.

Please note that any revision to previously approved materials must be approved by this committee prior to initiation. Please use the appropriate Amendment forms for this procedure.

All UNANTICIPATED PROBLEMS involving risks to subjects or others (UPIRSOs) and SERIOUS and UNEXPECTED adverse events must be reported promptly to this office. Please use the appropriate reporting forms for this procedure. All FDA and sponsor reporting requirements should also be followed. All NON-COMPLIANCE issues or COMPLAINTS regarding this project must be reported promptly to this office.

Please note that all research records must be retained for a minimum of seven years after the completion of the project.

If you have any questions, please contact the IRB Office at 301-405-4212 or irb@umd.edu. Please include your project title and reference number in all correspondence with this committee.

Generated on IRBNet

This letter has been electronically signed in accordance with all applicable regulations, and a copy is retained within University of Maryland College Park (UMCP) IRB's records.

Generated on IRBNet

UNIVERSITY OF MARYLAND
INSTITUTIONAL REVIEW BOARD

1204 Marie Mount Hall
College Park, MD 20742-5125
TEL 301.405.4212
FAX 301.314.1475
irb@umd.edu
www.umresearch.umd.edu/IRB

DATE:	January 19, 2023
TO:	Ernesto Calvo, PhD
FROM:	University of Maryland College Park (UMCP) IRB
PROJECT TITLE:	[1825785-8] COVID-19, Trust, and Misinformation
SUBMISSION TYPE:	Amendment/Modification
ACTION:	APPROVED
APPROVAL DATE:	January 19, 2023
REVIEW TYPE:	Expedited Review
REVIEW CATEGORY:	Expedited review category # 7. Waiver of Consent Documentation, 45CFR46.117(c). Waiver of Consent, 45CFR46.116(f)(3) (deception).

Thank you for your submission of Amendment/Modification materials for this project. The University of Maryland College Park (UMCP) IRB has APPROVED your submission. This approval is based on an appropriate risk/benefit ratio and a project design wherein the risks have been minimized. All research must be conducted in accordance with this approved submission.

Prior to final approval of this project scientific review was completed by the IRB Member reviewer.

This submission has received Expedited Review based on the applicable federal regulations.

This project has been determined to be a MINIMAL RISK project.

Please remember that informed consent is a process beginning with a description of the project and insurance of participant understanding followed by a signed consent form. Informed consent must continue throughout the project via a dialogue between the researcher and research participant. Unless a consent waiver or alteration has been approved, Federal regulations require that each participant receives a copy of the consent document.

Please note that any revision to previously approved materials must be approved by this committee prior to initiation. Please use the appropriate Amendment forms for this procedure.

All UNANTICIPATED PROBLEMS involving risks to subjects or others (UPIRSOs) and SERIOUS and UNEXPECTED adverse events must be reported promptly to this office. Please use the appropriate reporting forms for this procedure. All FDA and sponsor reporting requirements should also be followed. All NON-COMPLIANCE issues or COMPLAINTS regarding this project must be reported promptly to this office.

Please note that all research records must be retained for a minimum of seven years after the completion of the project.

If you have any questions, please contact the IRB Office at 301-405-4212 or irb@umd.edu. Please include your project title and reference number in all correspondence with this committee.

Generated on IRBNet

This letter has been electronically signed in accordance with all applicable regulations, and a copy is retained within University of Maryland College Park (UMCP) IRB's records.

Generated on IRBNet
